# Hunger modulates exploration through suppression of dopamine signaling in the tail of striatum

**DOI:** 10.1016/j.neuron.2025.09.009

**Published:** 2025-10-02

**Authors:** Tarun Kamath, Bart Lodder, Eliana Bilsel, Isobel Green, Rochelin Dalangin, Michelle Raghubardayal, Wengang Wang, Paolo Capelli, Jessie Legister, Joshua Timmins, Lauren Hulshof, Janet Berrios Wallace, Lin Tian, Naoshige Uchida, Mitsuko Watabe-Uchida, Bernardo L. Sabatini

**Affiliations:** 1 Howard Hughes Medical Institute, Department of Neurobiology, Harvard Medical School, Boston, MA 02115, USA; 2 Department of Molecular and Cellular Biology, Center for Brain Science, Harvard University, Cambridge, MA 02138, USA; 3 Present address: CERVO Brain Research Center, Laval University, Quebec, QC G1E 1T2, Canada; 4 Max Planck Florida Institute for Neuroscience, Jupiter, FL, 33458 USA.

**Keywords:** Exploration, dopamine, hunger, fluorescence lifetime imaging, tail of striatum

## Abstract

Caloric depletion induces behavioral changes that help an animal find food and restore its homeostatic balance. Hunger increases exploration and risk-taking behavior, allowing an animal to forage for food despite risks; however, it is unknown which neural systems coordinate such behavioral adaptations. Here, we characterize how hunger restructures an animal’s spontaneous behavior as well as its directed exploration of a novel object. We show that hunger-induced changes in exploration are accompanied by and result from modulation of dopamine signaling in the tail of the striatum (TOS). Dopamine signaling in the TOS is in turn modulated by hunger through the activity of agouti-related peptide (AgRP) neurons, putative “hunger neurons” in the arcuate nucleus of the hypothalamus that are polysynaptically connected to the TOS through the lateral hypothalamus. Thus, we delineate how hypothalamic systems modulate dopaminergic circuitry to mediate changes in exploration behavior in the hungry state.

## eTOC

Kamath et al. examine the neural mechanism by which hunger makes animals take more risks. They show that hunger neurons in the hypothalamus suppress threat and salience-related dopamine signals in the tail of striatum during bouts of novelty exploration and risk assessment.

## INTRODUCTION

Hunger promotes goal-directed behaviors that are thought to help an animal restore its homeostatic balance^1^. Food-seeking is one important behavioral correlate of the hungry state, but there are additional changes in behavior that are not obviously oriented towards obtaining caloric rewards, including decreased aversion of open spaces, increased tolerance for certain types of pain, increased risk-taking in the face of predation, and changes in territorial aggression^1^. Behavioral adaptation in the hungry state also includes increased exploration and risk-taking^2^. For example, calorically-depleted animals spend more time exploring mazes and novel stimulus^3–5^. Increased risk-taking and exploration are beneficial to finding new food sources and suppressing the aversion of novel stimuli can promote foraging. Although the neural circuits underlying many hunger-induced behavioral changes have been elucidated^3,4,6–9^, those involved in hunger-induced risk-taking are unknown.

Exploration is modulated by the activity of striatal-projecting midbrain dopaminergic neurons (DANs)^10,11^. The activity of midbrain DANs changes when animals receive novel information that is predictive of an upcoming reward, and when animals engage with novel stimuli that lack clear value^12,13^. Phasic dopamine (DA) transients in the nucleus accumbens and basolateral amygdala triggered by, respectively, overtly rewarding and aversive stimuli, are modulated by caloric state^14,15^. It is not known, however, if caloric state impacts dopaminergic responses to a salient, non-caloric stimulus which, in turn, regulates exploratory and novelty-seeking behaviors.

Agouti-related peptide (AgRP) expressing neurons in the arcuate nucleus of the hypothalamus (ARC) modulate food intake and feeding-related behaviors and are activated during fasting^16,17^. Here, we show that, consistent with previous studies, hunger increases novel object exploration. This increased exploration is mediated through suppression of DA signaling in the TOS, a nigrostriatal DA signaling pathway thought to represent salience and threat prediction^13,18–20^. Hunger increases the rate at which mice acclimate to a novel object in their environment, as seen both in behavioral desensitization and by experience-dependent suppression of TOS DA transients. The hunger signal that modulates novel object exploration is derived from the activity of AgRP neurons, which bidirectionally modulate exploration and associated TOS DA signaling and poly-synaptically project to the TOS through the lateral hypothalamus. Thus, we describe neural systems that concomitantly regulate exploration with caloric need.

## RESULTS

### Hunger restructures spontaneous, open-field exploratory behavior

**Figure 1:**
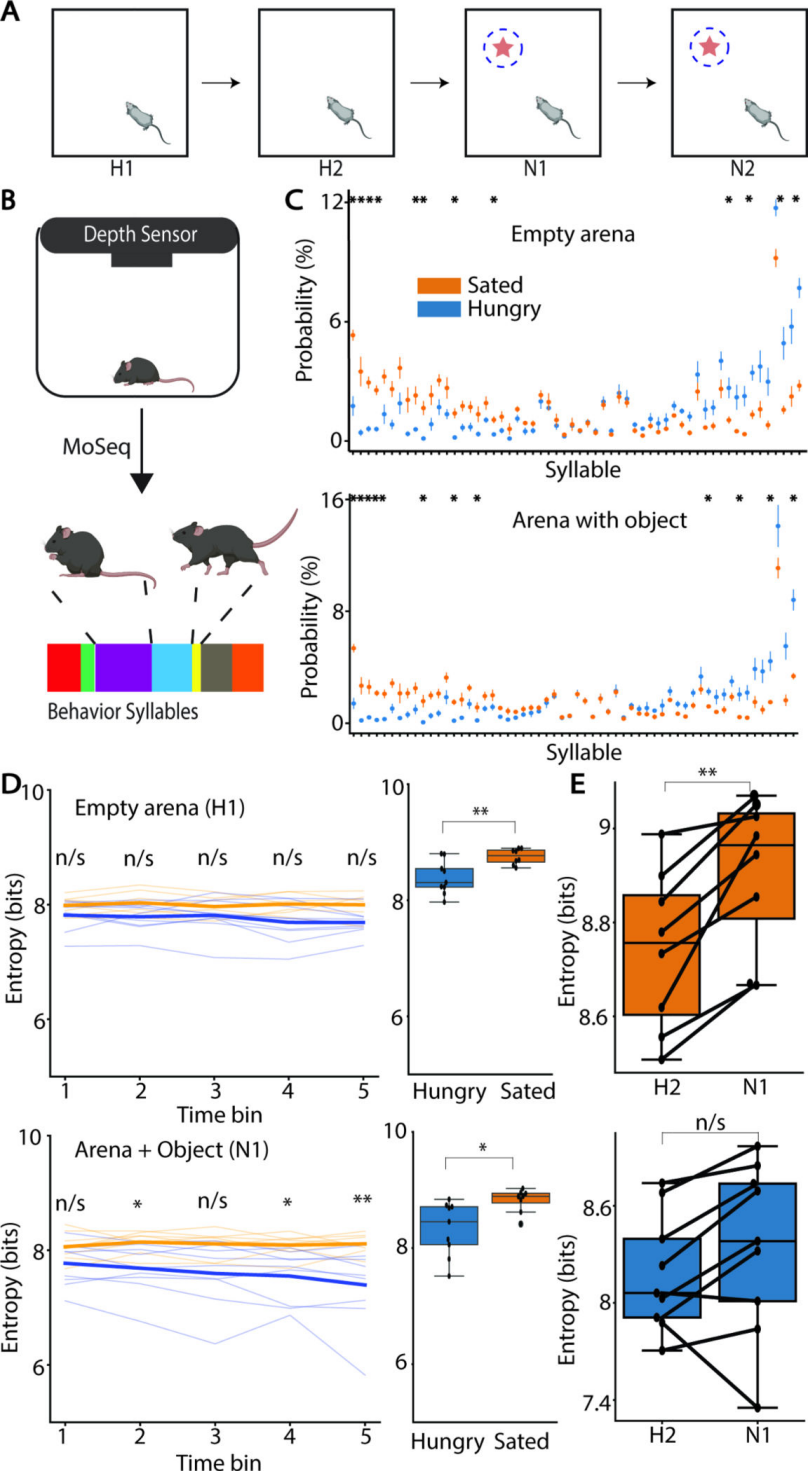
Hunger restructures spontaneous exploration behavior A) Schematic of the open-field NOE assay. Star indicates the novel object, dashed blue lines indicate interacting radius. B) MoSeq workflow to capture animal behavioral data and segment it into individual syllables. C) Changes in syllable frequency between hungry and sated animals when animal explores an empty arena (days H1-H2, top) or an arena with an object (days N1-N2, bottom). Values are normalized to total syllable usage across all animals in each type of session. (n=9 hungry, n=8 sated mice, two sessions/animal, *:p<.05, Mann-Whitney U-test with Bonferroni correction. Points represent the mean and error bars the SEM. D) Syllable transition entropy for animals during the first session without object present divided into quintiles (top left; n/s: p>.05, Mann-Whitney U-test with Bonferroni correction) and compared across groups (top right) averaged across sessions H1–2, and similar comparisons with the object present (bottom row; Mann-Whitney U-test with Bonferroni correction). E) Changes in syllable transition entropy between day 2 of exploring an empty arena to day 1 of exploring arena with object for sated (top) and hungry (bottom) animals.

To study how hunger modulates exploration, we used a two-stage, open-field novel-object exploration (NOE) assay in which mice were either food-restricted (hungry) or given food *ad libitum* (sated) and allowed to freely explore an empty arena for two days before an object is placed in one section of the arena (Figure 1A)^18^. Mouse movements and posture were captured with an overhead camera and analyzed with Motion Sequencing (MoSeq), an unsupervised algorithm that segments continuous animal behavior into characteristic, discrete “syllables” (Figure 1B)^21,22^. Hungry and sated animals employ behavioral syllables at different frequencies in their spontaneous behavior in both types of sessions (Figure 1C; Table S1), consistent with state-dependent changes in behavior ^23,24^.

MoSeq describes continuous behavior as a Markovian process, allowing for analysis of the statistics of syllable transitions such as the distribution of the frequency of transitions between pairs syllables (e.g., how frequently does the animal transition from syllable A to B). The entropy of the syllable transition distribution reflects the stereotypy of syllables sequences: more stereotyped behavior has lower entropy than does random behavior^22^. The syllable transition entropy of hungry animals is lower than that of sated animals both in the presence (U=64, p=0.0055) and absence (U=62, p=0.011) of the object (Figure 1D). Thus, hungry mice exhibit less variability in the sequence of syllables used during spontaneous movements.

Mice explore novel stimuli and the addition of a novel, salient object in the environment changes their behavior. However, animals do not significantly change syllable usage with the introduction of the novel object (Figure S1A). The mouse interacts with the object only a minority of the time, potentially masking differences in syllable usage during these interactions. Indeed, a subset of syllables were preferentially expressed when mice where near the novel object (Figure S1B; Table S1). Of these, one syllable (syllable 50) was used at different rates by hungry and sated mice (Figure S1C right; Mann-Whitney U test with Bonferroni correction, U=3, p=0.029). Both sets of mice use syllable 50 less frequently as N1 session progresses, with use in hungry mice decaying more rapidly (Figure S1C; Friedman repeated measure test; hungry, W=0.171, p=2*10^-5^; sated, W=0.375, p=3.44*10^-12^). Additionally, the presence of the object decreases the behavioral structure (i.e., increased the entropy) of sated (Wilcoxon paired signed-ranked test; W=0, p=0.008), but not hungry (W=10, p=0.164), mice (Figure 1E). Thus, the novel object differentially restructures behavior in hungry and sated animals, indicating that hunger alters the impact that a novel object has on spontaneous behavior.

### Hunger changes exploration of a novel object in a familiar arena

**Figure 2:**
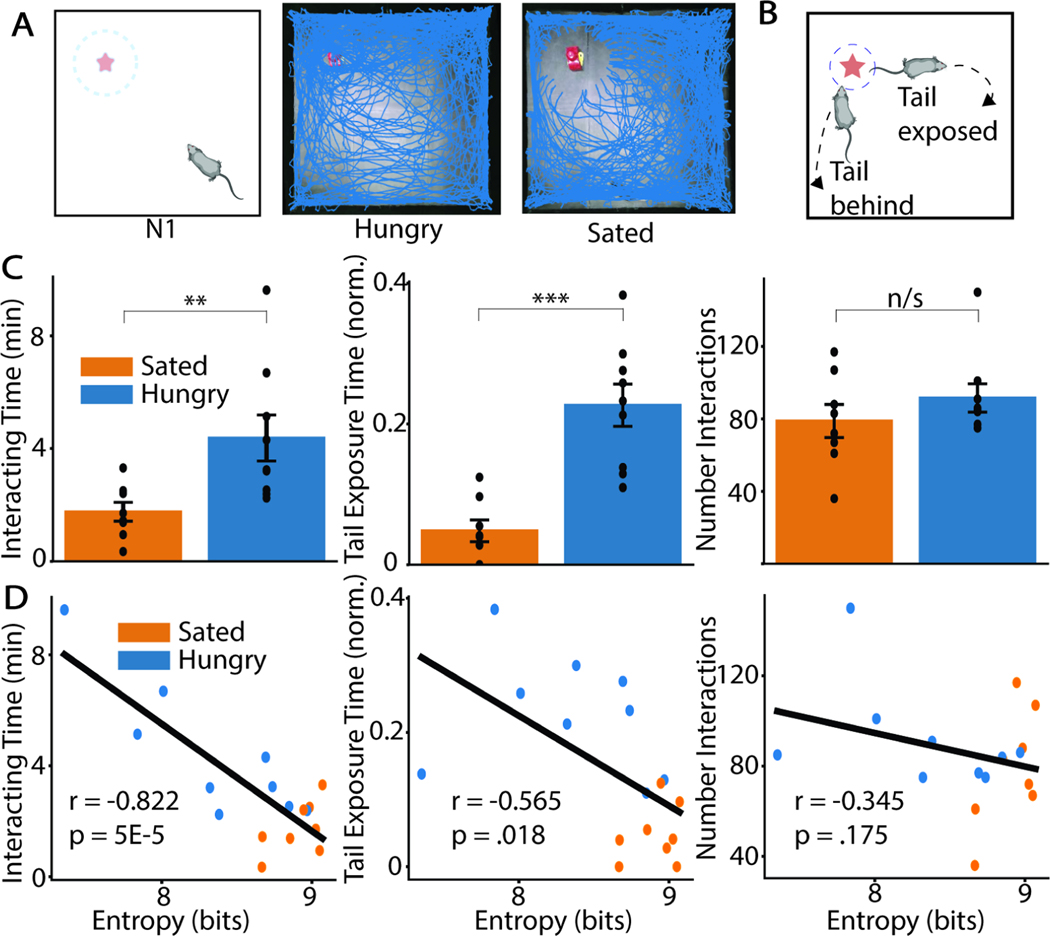
Hunger increases directed interaction and reduces risk assessment of a novel object **A)** Schematic of animal exploration of arena with a novel object present (left). Comparison of the position (red trace) of a hungry (middle) and sated (right) animal on the first day of exposure to the novel object. **B)** Mice retreat from a novel object either with tail behind the entire time (bottom left) or with their tail exposed (top right). **C)** Comparison of three novel object exploration statistics – left: total time spent near the object (Interacting Time), middle: fraction of the time the animal is around the object that their tail is closer than their nose (Tail Exposure Time norm.), and right: number of times the animal interacts with the object (Number Interactions). Height of bar and error bars show mean ± SEM. **D)** Regression (Pearson’s correlation coefficient) of novel object exploration metric and syllable transition entropy across animals on day N1.

MoSeq revealed differences between hungry and sated mice in overall behavioral structure as well as potentially in novel object investigation. To more deeply study the differences in novelty exploration between these two groups of animals, we employed keypoint tracking^25^. Mice explore novel objects using characteristic approach-avoid bouts^13^ which can be subdivided into those in which the animal either keeps its tail behind its nose the entire time (tail-behind bouts) or exposes its tail to the object (tail-exposed bouts) (Figure 2A-B). Switching between these modes of interaction is thought to reflect changes in risk perception, with tail-behind bouts resembling the “stretch-attend” posture adopted during risk assessment^26–30^.

We find that hungry animals (n=9) spend more time exploring a novel object than sated animals (n=8) (Interacting Time: U=63, p=0.008), and while interacting with the object, spend a greater fraction of time with their tail exposed to the object (normalized Tail Exposure Time: U=71, p=0.0009), but do not approach the object more often (Number Interactions: U=47, p=0.31) (Figure 2A,C). Furthermore, each animal’s syllable transition entropy on the first day of novel object exploration (day N1) is inversely correlated with how much the animal explores the object and how much it exposes its tail towards the object, but not with how many times the animal approaches the object (Figure 2D). A similar correlation is found between how much the animal explores the object and its behavioral structure even when accounting for group differences, indicating that the relationship between behavioral structure and object exploration is not driven entirely by differences between the groups of animals (Figure S1F).

### Hunger changes risk-assessment behavior in the presence of expected reward

A novel object is salient stimulus that induces changes in behaviors and neural activity associated with threat-perception in mice^13,18^ without causing adverse outcomes. To further understand the impact of hunger on risk-assessment behavior, we examined its effects on behavior in a threat-reward conflict assay (Figure S2A) ^20^. In this assay, mice were first trained to forage to collect caloric reward (Ensure droplet) from a delivery spout located far from a shelter. After training, half the animals were re-fed and then allowed to once again collect caloric rewards. The following day, a fictive predator (“monster”) was introduced, such that when the animal crossed an invisible line, the monster “charges” at the mouse, introducing highly salient noise and visual stimuli. The reward is positioned such that the animal can retrieve the reward without being harmed by the monster – thus the monster is a “threat” rather than a purely aversive stimulus.

When the monster was not present, hungry animals collected more caloric rewards than sated animals (Figure S2B; U=58, p=0.036; n=9 hungry, n=8 sated animals) and did so earlier in the session (Figure S2C; U=14, p=0.028). Introduction of the monster reduced the number of rewards collected by sated mice to nearly zero. Hungry mice collected collected more rewards (Figure S2B; U=60, p=0.008) and with less delay (Figure S2C; U=12, p=0.008) than sated mice despite the presence of the monster. In the absence of the monster, hungry and sated mice spent the same amount of time exploring the foraging arena per bout (Figure S2D; U=38, p=0.888) whereas, upon introduction of the monster, sated animals spent significantly less time in the arena than hungry animals (Figure S2D; U=62, p=0.011). These results indicate that hunger modulates threat assessment behavior in the presence of caloric reward.

### Dopamine transients in the TOS are modulated by hunger

The mesostriatal dopaminergic system regulates both novel-object exploration behavior and behavioral structure^10,18,31^. In particular, DA is released in the TOS when mice retreat from a novel object. The magnitude of this increase is inversely correlated with the amount the animal explores a novel object, supporting that TOS DA release reflects a threat prediction error that guides learning about potential threats in the environment^18^. We hypothesized that increased novel object exploration in hungry animals is caused by reduced phasic TOS DA signaling.

**Figure 3:**
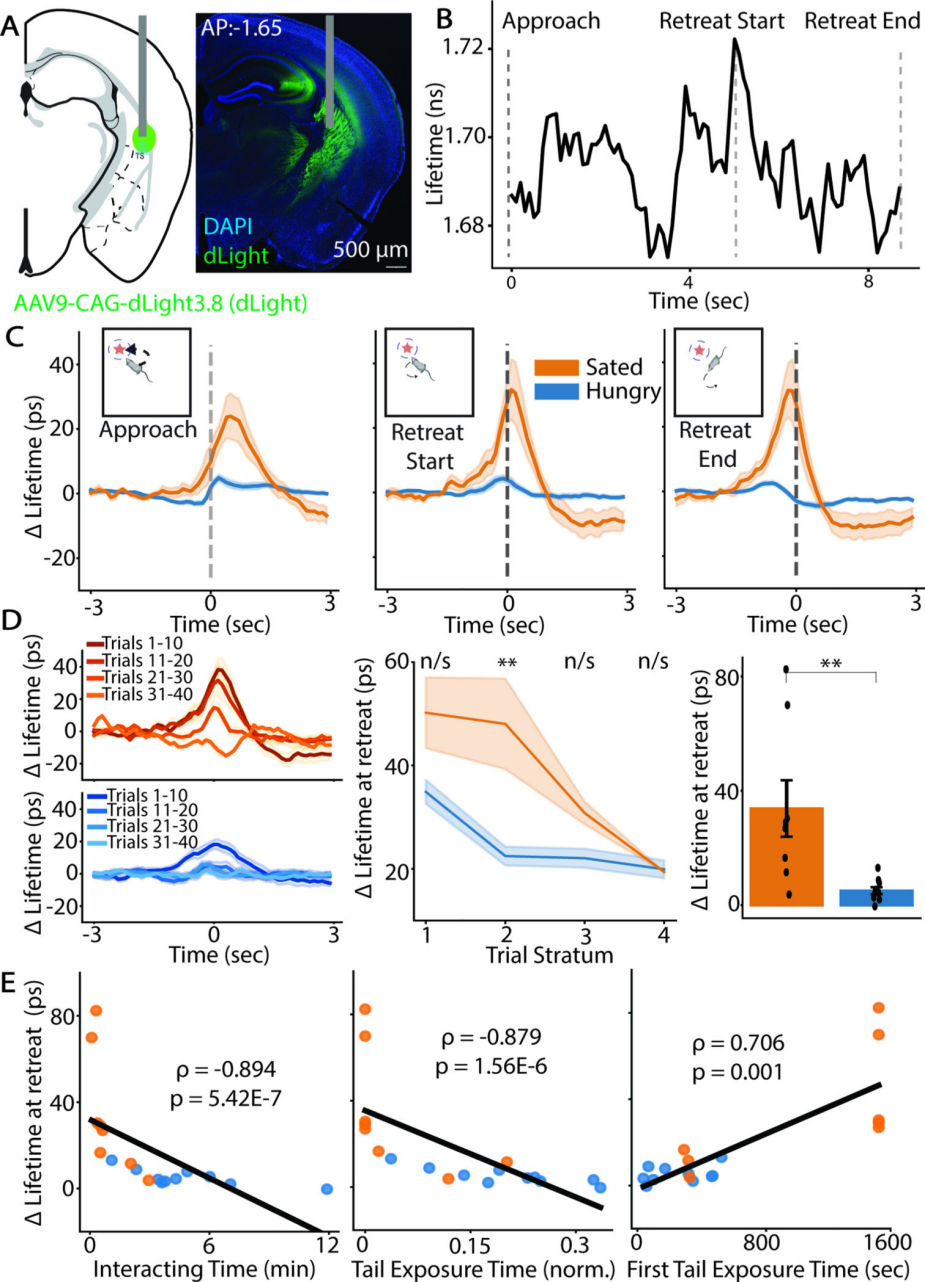
Hunger decreases dopamine release in the tail of the striatum during novel object assessment A) Schematic of injection/surgery in the TOS (left) and an example histology of fiber placement (right). Gray bar indicates fiber optic. B) dLight3.8 fluorescence lifetime fluctuations in the TOS when animals interact with a novel object. C) Hunger modulation of dLight3.8 fluorescence lifetime fluctuations when animals interact with a novel object. Graphs are aligned to when an animal approaches the novel object (Approach), begins to move away it (Start Retreat), and stops interacting with it (End Retreat). Bold line and shaded area represent mean and SEM across animals. This is true for all photometry graphs unless otherwise specified. D) Depression of dLight3.8 fluorescence lifetime fluctuations as animals make repeated interactions with the object (“trials”). Signals are grouped into strata of 10 trials for sated and hungry animals (left). The change in the magnitude of the retreat-associated lifetime signal across these strata (middle; Mann-Whitney U-test with Bonferroni correction, *:p<0.05), and the difference in the magnitude of the retreat-associated lifetime signal between hungry and sated animals (right). Bar and error bars: mean ± SEM. E) Regression (Spearman’s rank correlation coefficient) of the dLight3.8 lifetime signal during retreat for an animal with its exploration of a novel object.

To test this hypothesis, we used adeno-associated virus (AAV) to express a genetically-encoded DA sensor, dLight3.8 (Roshgadol and Chouinard et al., in preparation), in the TOS and implanted an optic fiber to record DA-dependent changes in fluorescence (Figure 3A, S4A). Upon ligand binding, dLight3.8 exhibits robust increases in fluorescence intensity and lifetime (Roshgadol and Chouinard et al., in preparation)^32^. Fluorescence lifetime is insensitive to many artifacts that plague intensity measurements such as differences in sensor concentration, fiber placement, photobleaching and hemodynamics^32–34 35^. Therefore, we used dLight3.8 fluorescence lifetime to measure fluctuations in DA concentration in the TOS using **f**luorescence **li**fetime **p**hotometry at high temporal **r**esolution (FLiP-R)^32,35^.

On the first day of the NOE assay, TOS dLight3.8 fluorescence lifetime signal rises, peaks, and then falls when a mouse, respectively, approaches, begins to retreat from, and fully retreats from the object (Figure 3B). dLight3.8 fluorescence lifetime transiently increases upon retreat from the novel object in both hungry and sated mice (Figure 3C). In sated, but not hungry, animals, the lifetime then transiently dips below baseline shortly after the start of retreat (Figure 3C). The transient increase in lifetime around the object decreases with repeated novel-object exploration bouts in hungry and sated animals (Figure 3D), consistent with habituation to the presence of the object. The magnitude of this retreat-associated lifetime signal (ΔLifetime at retreat) is comparable between hungry and sated animals in the first several interactions but decays more rapidly over object exploration bouts in hungry animals (Figure 3D, S3B).

Comparison of the magnitude of the retreat-associated dLight3.8 lifetime signal across all trials reveals that hungry, compared to sated, animals have smaller average retreat-associated lifetime transients (reflecting decreased DA release) in the TOS (U=7, p=0.002, n=10 hungry, n=8 sated animals) (Figure 3D). Analysis of interactions in which animals did not expose their tails also revealed decreased TOS DA signal in hungry compared to sated animals (U=11, p=0.008) (Figure S3C). Moreover, although hungry animals spend longer exploring the object (U=82, p=0.012), the speed at which they retreat from the object is not significantly different than that of sated animals (U=19, p=0.068) and trial-by-trial variance in the speed of retreat does not explain variance in TOS DA signals (t=-1.912, p=0.073) (Figure S3D-E). Thus, differences in the DA transients are not driven by gross behavioral differences between the two groups of animals. Fluorescence lifetime transients were not appreciated in mice expressing a ligand-binding mutant version of dLight3.8, confirming that the lifetime fluctuations are not driven by ligand-independent effects (Figure S3F). Thus, these results suggest that changes in novel-object exploration behavior in hungry animals may result from modulation of TOS DA signaling.

TOS DA is typically modulated by salient external events (e.g., loud sounds, lunging objects) but not by reward predicting objects (e.g., water)^13,20^. However, it is unknown how TOS DA is modulated by the presence of an *unexpected* caloric object. Therefore, we examined TOS DA in animals that were habituated to a small arena and then were randomly delivered chocolate pellets (Figure S3G). Although animals had been pre-exposed to the pellets on the day before the assay, the presence of the pellets was new to the arena, thus constituting an “unexpected familiar” stimulus^18^. We found that TOS dLight3.8 fluorescence lifetime increases upon retrieval and consumption of the pellet (Figure S3G). Repeated exposure to pellets did not change the magnitude of this DA signal (Friedman test, W=0.159, p=0.766).

### TOS DA transients modulate novel object exploration in hungry animals

**Figure 4:**
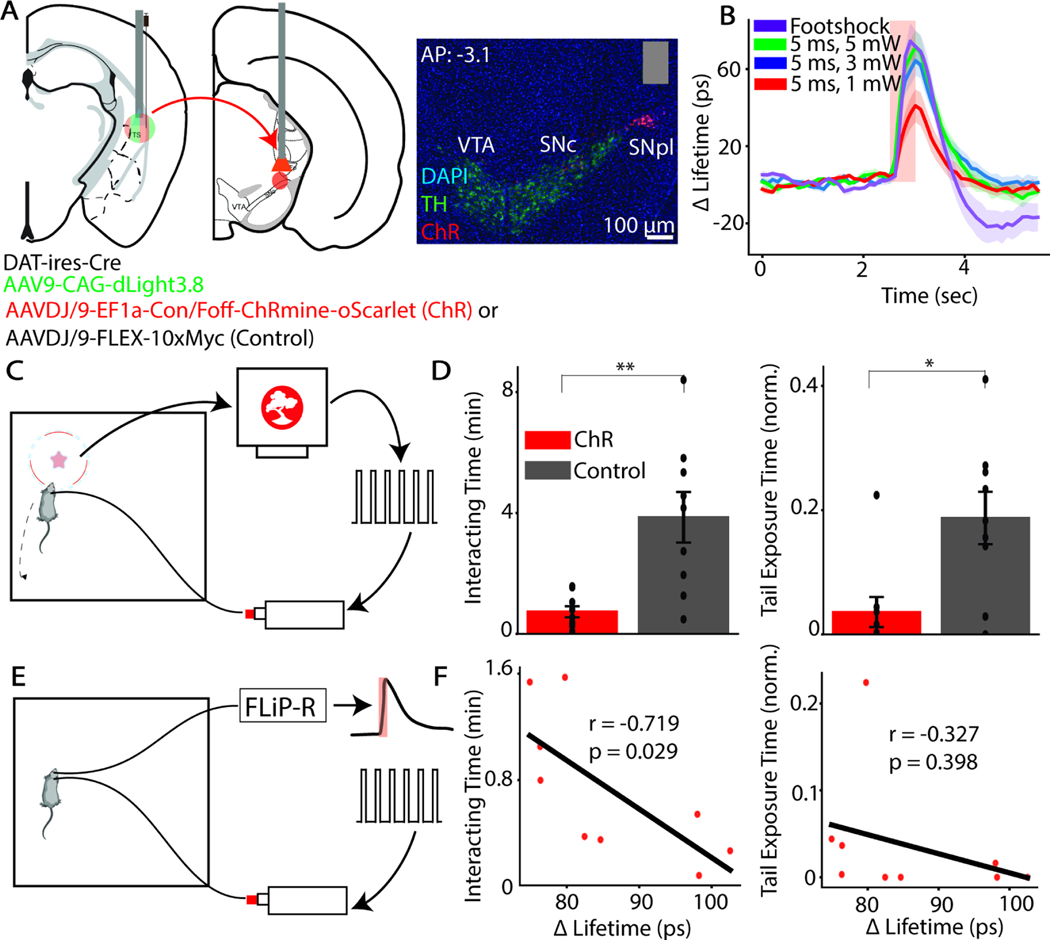
TOS DA modulates novelty exploration and risk assessment behavior in hungry animals **A)** Injection and surgery schematic for manipulating TOS-projecting dopaminergic neurons and recording and resulting release (left) and example histology placement of stimulating fiber in the midbrain (right). Gray bar indicates fiber optic placement. **B)** Calibration of optogenetic stimulation parameters to evoke similar dopamine release as a foot shock. Red bar represents time of either optogenetic stimulation or foot shock. **C)** Schematic of closed-loop optogenetic activation of TOS-projecting DANs when animal approaches a novel object (star). The animal’s presence within a given ROI around the object (red dashed circle) is processed in real-time using the Bonsai-rx software (top computer), which then generates a pulse train (right) to allow for pulsed activation of a laser (bottom), which is transmitted to the mouse. **D)** Effect of optogenetic activation of TOS-projecting DANs in hungry mice on novel object exploration behavior. Height of bar and error bars represent the mean ± SEM. **E)** Schematic of post-hoc validation of evoked DA release in the TOS with optogenetic stimulation using FLiP-R in a separate open field arena without the object present. The same optogenetic pulse pattern as was used in the behavioral assay was fed into a laser (right pulse pattern and laser). Simultaneously, on the ipsilateral hemisphere, fluorescence lifetime signal was collected from the fiber implanted in the TOS to record fluorescence lifetime fluctuations in the TOS (left mouse). The signal was generated by the FLiP-R system (top) and analyzed post-hoc (right trace with overlying red rectangle representing the time of optogenetic activation.) **F)** Regression (Pearson’s correlation coefficient) of change in exploration and risk assessment with level of DA release evoked by the optogenetic stimulation parameter across mice.

To determine if the hunger-induced changes in TOS DA signaling cause the change in NOE behavior, we examined if optogenetically stimulating TOS DA release during interactions with a novel object decreases its exploration. To target TOS-projecting DANs, we employed a retrograde strategy using a serotype of AAV, AAVDJ/9, that infects DAN axons (Figure S4A)^36^. In DAT-*ires*-Cre mice, we bilaterally co-injected two viruses in the TOS: (1) Cre-dependent AAVDJ/9 to express ChRmine^37^ in DANs and (2) AAV to express Cre-independent dLight3.8 in TOS. In control animals, the Cre-dependent ChRmine construct was replaced by a non fluorescent control protein (10xmyc) (Figure 4A). We implanted fiber optics in TOS and over SNpl (the primary site from which TOS-projecting DANs originate) (Figure 4A) for simultaneous stimulation of DAN cell bodies via the SNpl fiber and measurement of resulting TOS DA release via the TOS fiber. TOS-projecting DANs do not substantially collateralize outside of the TOS; thus, this manipulation relatively specifically evokes release of DA in this striatal subregion (Figure S4B)^13^.

We calibrated the optogenetic stimulation to mimic the DA release evoked by a natural stimulus. Since DA release in the TOS is modulated by threatening stimuli, we exposed an initial cohort of hungry animals to a foot shock, a robustly threatening/aversive stimulus, to drive DA release in the TOS (Figure 4B). We identified optogenetic stimulation parameters that evoked TOS DA release of similar amplitude to the foot shock (n=4 sites across n=3 animals) (Figure 4B).

To perform closed-loop manipulation of TOS DA signaling during behavior, hungry animals were allowed to explore the novel object as their position was tracked in real-time^38^. When a portion of the animal’s body fell within 7 cm of the center of the object (~4–5 cm from its edge), the optogenetic stimulation was delivered (Figure 4C). ChRmine expressing animals (n=9), under closed-loop optogenetic activation of DANs, spend less time interacting with the object (U=74, p=0.004) and a smaller fraction of that time with their tail exposed towards the object (U=58, p=0.016) than control hungry animals (n=9) experiencing the same stimulation paradigm (Figure 4D). The magnitude of the optogenetically-evoked DA signal is similar to that seen when a sated animal initially retreats from the novel object (Figure S4C; U=20, p=0.139; n=9 opsin hungry animals, n=8 non-opsin sated animals). The reduction in exploration in hungry opsin-expressing mice is not driven by alteration of their food-seeking drive as these mice eat the same amount as control hungry mice in a feeding test conducted immediately after the NOE assay (U=5, p=0.689; n=9 control hungry animals, n=8 opsin experimental animals) (Figure S4D).

To understand whether variance in exploration in the opsin-expressing mice could derive from variance in the evoked TOS DA signal, we used FLiP-R to measure optogenetically-evoked DA release for each animal shortly following the NOE assay in a different open arena without the object present (Figure 4E). The level of evoked TOS DA release in the open field inversely correlates with time spent exploring the object (Figure 4F), suggesting that the TOS DA transients modulate exploration behavior in a graded manner. In contrast, although optogenetic activation of TOS-projecting DANs decreases tail exposure, the amount of tail exposure was not correlated with the magnitude of DA released (Figure 4F). Together, our data support that TOS DA release is important for hungry animals to learn about potential threats in the environment, and that stimulating this system is sufficient to cause hungry animals to avoid the novel object.

### AgRP neurons bidirectionally modulate NOE and associated TOS DA transients

**Figure 5:**
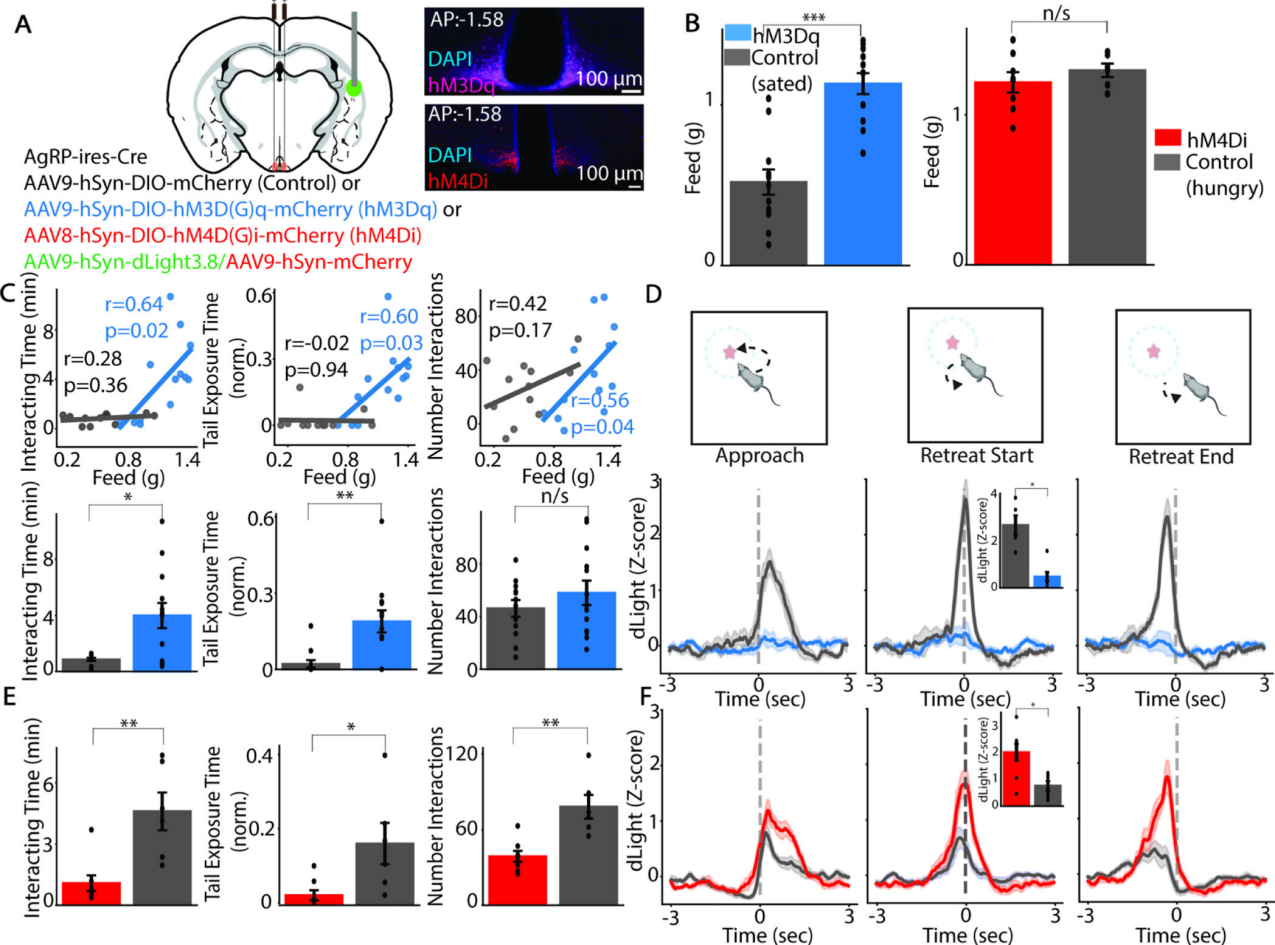
AgRP neurons modulate novelty exploration and risk assessment and associated DA fluctuations in the TOS A) Injection scheme and representative image for recording TOS DA and manipulating AgRP neurons with either excitatory or inhibitory DREADDs (left). B) Effect of hm3Dq-mediated AgRP neuronal excitation in sated animals on feeding behavior (left) and effect of hm4Di-mediated AgRP neuronal inhibition in hungry animals on feeding behavior (right). C) Regression (Pearson’s correlation coefficient) of effect of excitation of AgRP neurons in sated animals on feeding from panel B and exploration/risk assessment of a novel object (top). Effect of excitation of AgRP neurons on exploration/risk assessment of a novel object (left: Interacting time; middle: Tail Exposure Time norm.; right: Number Interactions) (bottom). Bar and error bars: mean ± SEM. D) Effect of excitation of AgRP neurons in sated animals on dLight3.8 fluorescence intensity fluctuations in the TOS during interactions with a novel object and comparison of retreat-associated TOS DA signal (inset, middle graph) E) As in panel C, except for inhibition of AgRP neurons in hungry animals. Height of bar and error bars represent the mean ± SEM. F) As in panel D, except for inhibition of AgRP neurons in hungry animals.

AgRP-expressing neurons in the ARC are activated by fasting and increased activity of these neurons decreases anxiety and increases risk-taking behavior^4,5,8,39,40^. AgRP neurons regulate DA release in the nucleus accumbens via a polysynaptic projection through the lateral hypothalamus^14,41^. We hypothesized that activity of AgRP neurons modulates exploration and TOS DA signaling. Thus, to test the contribution of AgRP neural activity in hunger-induced modulation of exploration and TOS DA, we perturbed the activity of AgRP neurons using designer receptors exclusively activated by designer drugs (DREADDs). In a subset of mice, we also recorded resulting changes in TOS DA signaling. To simultaneously manipulate AgRP neurons and record DA signaling, we utilized AgRP-*ires*-Cre mice injected with (1) a Cre-dependent AAV in the ARC to express either excitatory (hM3Dq) or inhibitory (hM4Di) DREADDs in AgRP neurons and (2) a Cre-independent AAV in the TOS to express dLight3.8. We implanted a fiber optic in the TOS to perform frequency-modulated intensity fiber photometry to measure TOS DA fluctuations (Figure 5A).

We hypothesized that stimulation of AgRP neurons in sated mice would increase both an animal’s feeding behavior and its exploration of a novel object. Chemogenetic activation of AgRP neurons in sated mice robustly increases feeding (U=147, p=1.9*10^-4^; n=12 control sated, n=13 hM3Dq experimental animals), the amount of time spent exploring the novel object (U=120, p=0.023), and the fraction of time spent with its tail exposed towards the object (U=129, p=0.004) (Figure 5B-C). AgRP neuron activation does not affect the number of times an animal approaches the object (U=89, p=0.567) (Figure 5C), similar to the behavioral phenotype in calorically depleted mice (Figure 2C). The effect sizes of the chemogenetically-induced feeding and chemogenetically-induced change in exploration are correlated animal-by-animal (Figure 5C), consistent with variability in transfection of AgRP neurons with the stimulatory DREADD influencing both food intake^16,42^ and exploration behavior. AgRP neuron stimulation also decreased the amplitude of dLight3.8 fluctuations in the TOS when animals retreated from the novel object (Figure 5D; U=1, p=0.005; n=5 control sated, n=7 hM3Dq animals), similar to caloric depletion (Figure 3C-D).

In contrast, inhibition of AgRP neurons does not change feeding in food-restricted animals (Figure 5B; U=22, p=0.445; n=6 control hungry, n=8 hM4Di experimental animals). In line with prior studies, inhibition of AgRP neurons decreases feeding in sated animals at the beginning of the dark cycle (Figure S5E; n=6 hM4Di experimental animals, Wilcoxon paired signed-ranked test; W=0, p=0.031) when animals tend to eat the most, confirming the efficacy of the chemogenetic manipulation^17,43^. Inhibition of AgRP neurons decreases exploration of the novel object (U=40, p=0.004), tail exposure towards the object (U=38, p=0.018), and the number of interactions with the object (U=40, p=0.004) (Figure 5E). Furthermore, the decreased exploration is accompanied by increased TOS DA signaling upon retreat from the novel object, (Figure 5F; U=7, p=0.029). Notably, control hungry animals did display a phasic TOS DA response upon retreat from the object, possibly from the stress of the intraperitoneal injection of designer drug delivered shortly before the behavioral assay. Bidirectional modulation of AgRP neural activity does not change average speed in the arena (hM3Dq: U=53, p=0.182; hM4Di: U=23, p=0.949) (Figure S5B, D), consistent with a lack of gross effects on locomotion.

### AgRP neurons polysynaptically project to the TOS

**Figure 6:**
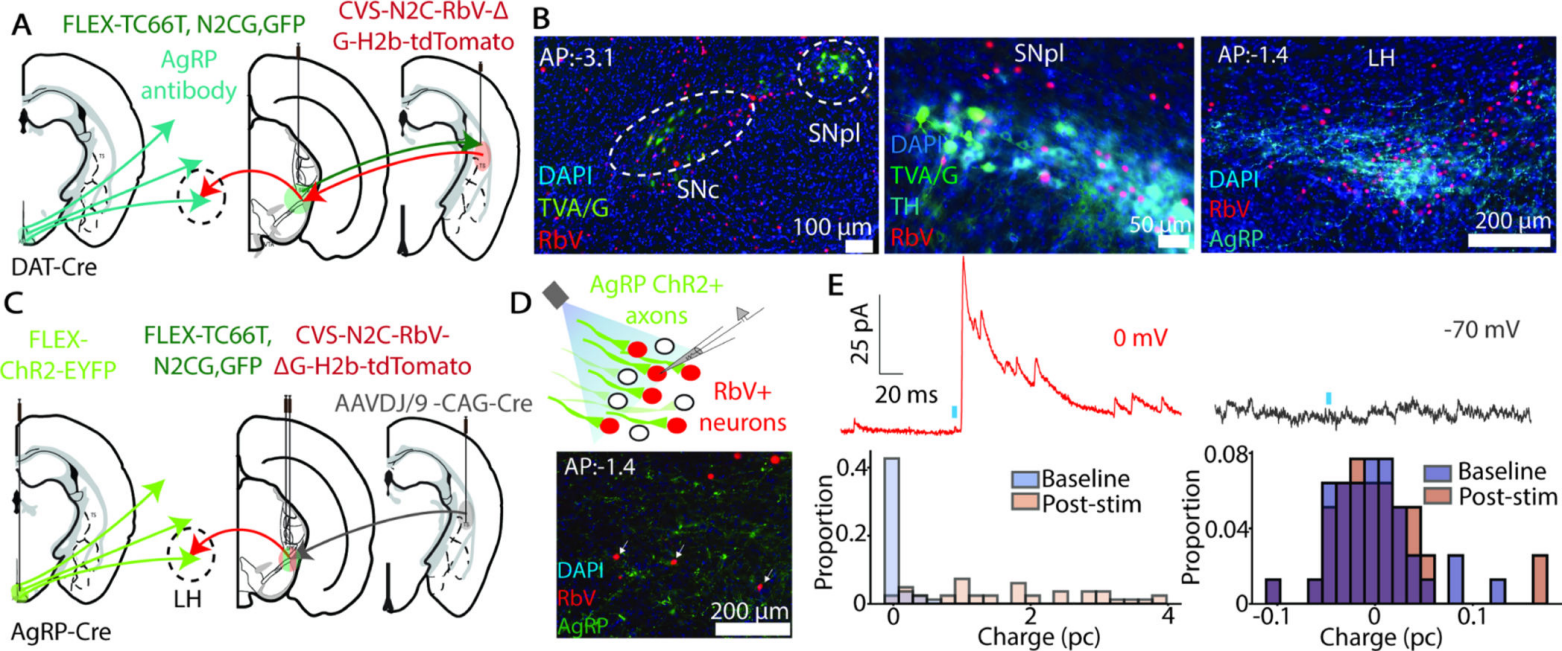
AgRP neurons polysynaptically project to the TOS through the lateral hypothalamus **A)** Schematic of rabies tracing experiment to map the intersection of AgRP projection fields and inputs to TOS-projecting DANs. **B)** Starter population of DANs in the SNpl that are positive for both TVA/G-protein (green) and RbV (red) (left) and confirmation that starter population are TH+ positive (right). **C)** Schematic of CRACM experiment to test synaptic link between AgRP neurons and the TOS. **D)** Schematic of whole-cell voltage-clamp recording used to record optogenetically evoked postsynaptic currents in RbV+ neurons (top) and location at which physiology recordings were conducted (bottom). **E)** Effect of full-field illumination and activation of ChR2+ axons on total charge of putative post-synaptic partners when cells are held at 0 mV (left; top is example trace, bottom is distribution of the pre vs. post total charge) and when cells are held at −70 mV (right).

To uncover the neuronal circuitry by which AgRP neurons may modulate TOS-projecting DANs, we performed projection-specific rabies virus tracing of monosynaptic inputs to TOS-projecting DANs. For this experiment, we used animals that express Cre recombinase under control of the dopamine transporter (DAT) promoter (DAT-*ires*-Cre)^44^. We injected an AAV carrying a Cre-dependent bicistronic construct encoding both a weakened EnvA receptor, TVA TC66T ^45^ and G protein in the substantia nigra of DAT-*ires*-Cre mice, causing expression of TVA and G proteins specifically in midbrain DANs. Four weeks later, we injected animals with EnvA-pseudotyped and G-deleted rabies virus (RbV) of the lower-toxicity CVS-N2C strain (CVS-N2C-RbV-ΔG-H2b-tdTomato) in the TOS ^46^. The RbV(EnvA) selectively infects TC66T-expressing DAN axons in the TOS, and once in the DANs, RbV are amplified in DANs that have been complemented by G, and transmit to neurons presynaptic to TOS-projecting DANs (Figure 6A). After waiting six days, we sacrificed animals and immunohistochemically stained for AgRP and examined the colocalization of AgRP+ axons and RbV+ neurons across brain regions (Figure 6A). As expected, primary infection (RbV+/TVA+) is largely restricted to DANs in the SNpl (Figure 6B).

There are three primary sites of colocalization between AgRP+ axons and retrogradely-labelled RbV+ neurons: the lateral hypothalamus (LH), the central amygdala (CeA), and the periaqueductal grey (PAG) (Figure 6B, S6A). In the absence of G protein, the pseudotyped RbV infects DAN axons in TOS and does not move beyond, confirming the necessity of G-complementation (Figure S6B). There is minimal colocalization of AgRP axons and inputs to TOS-projecting DANs in other major projection sites of AgRP neurons (Figure S6C).

To functionally examine synaptic connections in these pathways, we performed a channelrhodopsin-assisted circuit mapping (CRACM) experiment to link AgRP projections to TOS-projecting midbrain DANs. We used AgRP-Cre mice and injected Cre-dependent channelrhodopsin in the ARC, leading to expression of channelrhodopsin in AgRP neurons and axons. We also employed a retrograde strategy to target SNpl neurons which project to the TOS by injecting AAVDJ/9 encoding Cre recombinase (AAVDJ/9-CAG-Cre). The AAV efficiently infects axons of afferents to the TOS (including SNpl DANs) to express recombinase in TOS projecting neurons. We also injected mice in SNpl with the Cre-dependent bicistronic construct encoding TC66T and G protein to express these proteins selectively in TOS-projecting SNpl neurons. After waiting for channelrhodopsin and helper protein expression, we injected mice with CVS-N2C-RbV-ΔG-H2b-tdTomato in the SNpl. The rabies virus only infects and is amplified in TOS-projecting SNpl neurons and moves monosynaptically retrograde, labeling inputs to the TOS-projecting SNpl neurons (Figure 6C).

After waiting 5 days for RbV complementation and retrograde transsynaptic movement, we sacrificed animals and performed whole-cell voltage-clamp recordings to investigate potential synaptic connections between AgRP neurons and inputs to TOS-projecting SNpl neurons (Figure 6D). Brief optogenetic activation of AgRP+ axons evoked an inhibitory post-synaptic current (IPSC) in a subset of RbV+ cells in the LH when neurons were held at 0 mV (n=3/13 cells, N=2 animals; Figure 6E). No synaptic currents were detected when neurons were held at 70 mV (n=5 cells, N=2 animals; Figure 6E). The same protocol does not evoke IPSCs in RbV+ cells in the PAG (n=0/14 cells, N=3 animals; Figure S6D-E). Synaptic connections were not tested in the CeA given sparsity of AgRP+ axons at this site.

## DISCUSSION

In this study, we describe neural systems that underly changes in exploration behavior in hungry animals. We find that phasic DA signals in the TOS evoked when animals interact with a novel, *non-caloric* object are modulated by hunger in a manner that underlies hunger-dependent changes in novel object exploration. The hunger-dependent signal that regulates TOS DA signaling requires the activity of AgRP neurons in the ARC, which are polysynaptically connected to the TOS through the LH.

### Hunger modulation of spontaneous and directed exploratory behavior

Hunger-dependent changes in animal behavior have largely been studied in environments where food was once present (e.g., an animal’s home environment) or in the presence of food itself ^3,4,17,39^. In these instances, the animal may have a strong belief state about the value of the environment, which restructures behavior. Additionally, during the course of a behavioral assay in which animals are granted caloric rewards, the animal’s hunger state changes rapidly, making it difficult to ascertain whether changes in the hunger state of the animal or learning-related effects mediate changes in the animal’s behavior and neural signals. Instead, we investigated how hunger changes behavior in the absence of overt food cues. The animal’s hunger state is roughly constant during the short (~25 minutes) behavioral assay, which lacks food rewards; thus, behavioral changes during the session are due to other dynamic processes such as learning about whether the object is threatening.

We find that hunger increases the structure of spontaneous behavior, increases directed exploration and decreases risk assessment of a salient, novel object, metrics which are correlated on a per-animal basis. The more structured behavior in hungry animals is consistent with increased spatial exploration using a strategy that efficiently explores physical space with repetitive, stereotyped movements (e.g., spiral search)^47^. In contrast to prior results^17^ we do not observe hunger-evoked changes in average speed, potentially due to the lack of food-signaling cues in our experiments.

Hunger additionally decreases risk-assessment in a threat-reward conflict task^20^. This is in line with prior studies showing that hungry animals are more willing to overcome a potential predatory threat to obtain food^9^. Moreover, TOS DA signaling influences an animal’s ability to overcome threats to collect rewards in this assay^20^. Thus, future studies will examine if hunger also modulates TOS DA when animals need to overcome an overt threat to collect a familiar food.

### Hunger modulation of a nigrostriatal dopamine signaling pathway changes novelty exploration behavior

DA transients in the TOS may signal threat prediction errors (in contrast to those in the ventral striatum which may signal reward prediction errors^48^), such that lesions of TOS-projecting DANs decrease risk assessment behavior and allow animals to more quickly overcome threats in pursuit of perceived reward^13,18,20^. TOS DA is not as strongly modulated by rewards or reward predicting cues as is DA in the nucleus accumbens and dorsal striatum^48,49^. The TOS DA response integrates features of the salient stimulus that evoke the DA release^13,20^. We find that TOS DA signals integrate an animal’s internal state, such that hungry animals have decreased DA release in the TOS when interacting with a novel, non-caloric object. We propose that hunger facilitates exploration in the face of potential threat by suppressing DA-dependent threat estimates in the TOS. Similar fasting-induced decreases in DA release occur in the basolateral amygdala in response to an aversive tail shock in mice^15^. Fasting-induced changes in dopaminergic signaling have also been observed in *Drosophila melanogaster*, in which neurons expressing neuropeptide F (dNPF, which is related to hunger signaling in flies) modulates the activity of a subset of mushroom-body projecting DANs^50^. Fasting decreases the baseline activity of these DANs, while increasing the DA response to an aversive shock, potentially as a mechanism to maintain aversive learning in hungry flies^51^.

Hunger modulates several neural systems involved in anxiety and pain processing^6,8^. Moreover, TOS DA signaling is modulated by some, but not all, aversive and salient stimuli^13^. Thus, there are multiple neural circuits that may coordinate changes in NOE in hungry animals beyond TOS DA signaling. Nevertheless, closed-loop, calibrated optogenetic stimulation of TOS-projecting DANs in hungry animals is sufficient to reduce novel object exploration. Importantly, the variance in exploration behavior is correlated with the level of optogenetically-evoked TOS DA release. Under the framework that the TOS DA serves as a threat prediction error signal, the optogenetically-evoked DA signal drives decreased exploration in hungry animals due to fear that the object may produce an aversive outcome. Optogenetically-evoked TOS DA release does not drive avoidance in a real-time preference assay, suggesting that TOS DA alone is not an intrinsically aversive stimulus^52,53^. Coupled with our finding that hunger suppresses novel-object-evoked TOS DA responses, we propose that changes in NOE in hungry animals are due to modulation of TOS DA signaling.

Previous studies that optogenetically manipulated TOS DA did not calibrate the evoked DA release to match a behaviorally evoked signal^13,52–54^. We combined the AAVDJ/9 serotype to specifically target TOS-projecting DANs with FLiP-R to calibrate the optogenetic stimulation parameters to mimic DA release in the TOS evoked by a foot shock. Since novel object-associated TOS DA signals rapidly decrease as sated animals explore the object, it was not possible to calibrate the optogenetic stimulation to match the potential retreat-associated TOS DA signal in individual animals. However, the magnitude of optogenetically-evoked DA release matches the magnitude of signals observed in the first several retreats in sated animals. Thus, the optogenetic manipulation recapitulates physiologically- and behaviorally-relevant dopaminergic transients. This calibration was necessary as supraphysiological levels of evoked DA release may cause different behavioral effects from physiological levels of DA release^55–58^.

Hunger-induced changes in TOS DA are likely independent from the hunger-induced potentiation of DA signaling in the nucleus accumbens when animals receive food^14^. This is because the modulation of TOS DA occurs even when animals have not received food or encountered food in the environment and thus have no strong belief state that the environment may contain caloric rewards. This modulation of neural systems controlling risk assessment can be useful in natural foraging environments of wild mice which may span up to a half an acre and thus change rapidly^59^. In our NOE assay, the animal did not receive a caloric reward, but the hungry animals still explore the object; thus, hunger enhances exploration and risk-taking even in the absence of caloric reinforcers.

### AgRP neural activity relays caloric state information polysynaptically to change TOS DA and novelty exploration behavior

Hunger increases the magnitude of DA response in the nucleus accumbens to food through the activity of AgRP neurons, which disynaptically project to VTA DANs through the LH^14,41,60^. Activation of AgRP neurons increases feeding and exploration of a novel object. These two measures are correlated animal-by-animal, suggesting that AgRP neural activity is causal for the hunger-induced changes in exploration. This causality is established by bidirectional manipulation of AgRP neurons which alters both NOE and concomitant TOS DA signaling. The AgRP-induced suppression of TOS DA release in the NOE assay is opposite from the AgRP-induced potentiation of DA release in the nucleus accumbens in response to food, indicating that internal state modulation of DA signaling depends on the nature of the stimulus evoking DA release as well as the striatal subregion in which it occurs^14^.

Inhibition of AgRP neurons has a more subtle effect on exploration and TOS DA signaling than activation of these neurons, and does not change post-fast refeeding, potentially due to the ability of activity in a small fraction of AgRP neurons to coordinate aspects of hunger and feeding^16^. Chemogenetic inhibition of AgRP neurons similarly subtly modulates the activity of downstream neurons in the paraventricular hypothalamus^61^. Moreover, chemogenetic inhibition of excitatory inputs to AgRP neurons does not affect post-fast refeeding but does change homeostatic feeding in sated animals^62,63^. Thus, the chemogenetic manipulation may be insufficient to reduce strong activation of feeding behavior in fasted mice.

AgRP neurons trisynaptically project to the TOS through LH neurons that synapse onto TOS-projecting SNpl neurons. Since AgRP stimulation decreases DA signaling in the TOS and AgRP neurons inhibit LH neurons that connect to the TOS, we hypothesize that these intermediate LH neurons are excitatory. AgRP neurons form functional synapses with glutamatergic LH neurons^64,65^ and stimulation of glutamatergic LH neurons drives TOS DA release, suggesting that LH glutamatergic neurons provide direct input to TOS-projecting DANs^66^. Thus, AgRP neurons may reduce DA signaling in the TOS by decreasing the activity of LH excitatory inputs onto TOS-projecting DANs.

Our CRACM study is limited in two ways: first, the rabies-based tracing method labels inputs to all TOS-projecting SNpl neurons, not specifically DANs. GABAergic neurons in these midbrain regions also project to the striatum^57,67^. Second, we used short light pulses to assess fast neurotransmission; however, AgRP neurons also release neuropeptide Y and AgRP ^65^, which modulate the effect of inputs to downstream targets^43,61^. AgRP neurons may also regulate TOS-projecting DANs through an additional polysynaptic pathway^68^, or through neuropeptide release into the ventricles. Finally, although AgRP neurons are polysynaptically connected to the TOS through the LH, other pathways may transmit caloric state information to TOS-projecting DANs.

In summary, we identify how hypothalamic circuitry can modulate dopaminergic pathways to lead to ethologically adaptive increased exploration that accompanies hunger. This work sheds light on how dopaminergic circuitry integrates internal state to change how an animal learns to overcome threats and adapt its exploration even in the absence of reward.

## RESOURCE AVAILABILITY

### Lead Contact

Requests for further information and resources should be directed to and will be fulfilled by the lead contact, Bernardo Sabatini (bernardo_sabatini@hms.harvard.edu)

### Materials Availability

Newly generated plasmid constructs will be made available on Addgene (https://www.addgene.org/Bernardo_Sabatini/).

### Data/Code Availability

Data will be made available at Harvard Dataverse at the following link:https://dataverse.harvard.edu/dataset.xhtml?persistentId=doi:10.7910/DVN/FJGSZ5, Code for analysis will be made available at Github at the following link: https://github.com/bernardosabatinilab/Kamath_2025_Hunger. Any further information on data or analysis should be directed to and will be fulfilled by the lead contact, Bernardo Sabatini (bernardo_sabatini@hms.harvard.edu)

## STAR★METHODS

### EXPERIMENTAL MODEL AND STUDY PARTICIPANT DETAILS

Male and female mice aged 9–20 weeks were used. The following mouse lines were used: C57BL6/J (The Jackson Laboratory, 000664); *AgRP-ires-Cre* (The Jackson Laboratory, 012899); *DAT-ires-Cre* (The Jackson Laboratory, 006660). All transgenic mice were used as heterozygous and bred inhouse on a congenic C57BL/6J background. We did not observe any significant differences in behavior between the sexes and all behavioral experiments contained animals of both sexes. All behavioral studies used animals of both sexes but specific analysis of sex-specific differences was not conducted as the study was not powered enough to do so. Animals were group-housed until the day before experimentation began on a 12-hour reversed light/dark cycle with standard chow and water provided *ad libitum*. During behavioral experimentation, animals were single housed and for hungry animals, animals were food restricted to 80–90% of their *ad libitum* baseline weight. Behavioral tests were conducted between 12:00 and 19:00 each day, and animal behavior was assessed at roughly the same time day-by-day. All animal care and experimental procedures were approved by the Harvard Standing Committee on Animal Care following guidelines described in the US NIH Guide for the Care and Use of Laboratory Animals.

### METHOD DETAILS

#### Novel Object Exploration and Refeeding Assay

An open-field, freely-moving novel object exploration behavioral assay was implemented as described previously ^18^. A flat open field arena ~60 cm × 60 cm was used for experimentation. For behavioral experiments using Motion Sequencing, a black arena was lit with an overhead white light to visualize black mice on a black background. For all other experiments, the arena was white and illuminated with infrared LED strips. Briefly, the animals were habituated to handling for at least three days for 30 minutes per day. During handling, animals without implants or without injections were scooped using a transfer box. A transfer box was put into the corner of the animals’ cage and animals were allowed to freely enter it. Once the animal entered the box, the box was tilted up and replaced back on the floor of the cage. Handling ended once either 30 minutes had passed or the animal had entered the box five times ^18^. If animals had a fiber optic implant, handling instead consisted of briefly restraining the animals and manually attaching them to a fiber optic patch cord several times. Finally, if animals were included in DREADD manipulation cohorts, animals were habituated to intraperitoneal (IP) injections with saline every day of experimentation. After handling, animals were habituated to the empty arena for two days for ~25 minutes/day. During habituation, animals were placed into the arena from their cage and allowed to explore the arena spontaneously. Finally, on the novelty session days, a single novel object (a single Mega Blok, Mega Bloks First Builders 80-piece Classic Building Bag) was briefly submerged in soiled bedding mixed from each mouse’s cage in the current round and placed in the corner of the arena (~15 cm from either wall) ^18,72^. Animals were allowed to explore the arena as in habituation sessions for ~25 min. Behavior was recorded with an overhead camera (Kinect v2, Microsoft for Motion Sequencing; FL3-U3–13E4M, PointGrey for all other experiments). Video data was captured for MoSeq using publicly available recording software; all other video data was recorded using Bonsai-rx ^38^. Objects were cleaned with 70% ethanol at the end of each day and left out to dry overnight. The arena was cleaned with enzymatic cleaner (Nature’s Miracle) between animals every day and feces were removed. One object was used per animal and not reused across animals or cohorts. Animals were placed in the arena in the same order each day. For experiments with a feeding analysis, on the first novelty session day, after animals were removed from the arena, they were transferred to a recovery cage and given *ad libitum* food access. The amount the animal ate after three hours of food access was then recorded as the feeding amount for animals.

#### Motion Sequencing

Motion Sequencing (MoSeq) was implemented as described previously ^18,22^. The analysis steps for MoSeq are briefly as follows. The raw imaging data is pre-processed with filtering and background subtraction, and the animal’s outline and position were extracted. Principal components (PC) are then calculated to represent the movements of each mouse to reduce the dimensionality of the behavioral data. At this step, 600 frames were trimmed from the start of each video to exclude timepoints when the animal is not in the box. This data in PC space is then modeled using a non-robust, single transition matrix, auto-regressive hidden Markov model (AR-HMM) to segment the continuous behavior into individual syllables. We used MoSeq to divide the data into 100 syllables used across days without the object present (two sessions) and with the object present (two sessions). All code for extracting and modeling the data are available using the pipeline found here: https://dattalab.github.io/moseq2-website/index.html. The syllables that explained a cumulative 90% of the total frames across all the sessions were used ^73–75^, to a total of 58 syllables, for all analysis. Syllable ID numbers are arbitrary. Data from all behavioral sessions were used for syllable identification and initial modeling.

#### Syllable frequency analysis

Individual syllable frequencies were calculated across mice and across sessions for each type of session (empty arena, arena with object.) Syllable frequencies were normalized to type of session and syllables that are significantly enriched in one condition versus another (hungry versus sated animals) were identified using a Mann Whitney U test with Bonferroni corrections for multiple comparisons.

#### Entropy analysis

Entropy was calculated using the Shannon entropy formula (H =−Σ_i_𝜋_𝑖_log(𝜋_𝑖_)). Syllable transition entropy was calculated by generating a bigram transition matrix for each animal and session. Entropy was then calculated on this entire transition distribution, to model the randomness of the transition distribution on all transitions (including self-transitions). Entropy was calculated per session and animal and then averaged within animal and type of session (e.g. empty arena vs. arena with object present.) For rolling entropy analysis, a single session per animal was divided into quintiles and a separate transition matrix was built for each quintile and entropy calculations were made for those quintiles.

#### DeepLabCut analysis

To track the animal’s position relative to the object as well as quantify interactions with the object, we used DeepLabCut for pose estimation ^25^. We trained two separate networks for animals with fiber implants and for animals without fiber implants. We manually annotated the animal’s nose, head, and base of tail and trained a network on 20% of labeled frames. After using DeepLabCut to track these body parts in each video, we processed the files by first trimming those frames with <90% likelihood value, interpolating the animal’s position in between these frames, and smoothing the trajectory of the body part initially with a five-frame moving median filter (as recommended in DeepLabCut) and then post hoc with a 15-frame moving average filter. We defined the “interacting radius” as when one of the three body parts was within a 7 cm radius of the object. The beginning of a bout (start of approach) was defined as the earliest time of interactions to the time at which either the nose or the tail was within the interaction radius of the object. During each interacting timepoint, we determined whether the nose or tail was closer to the center of the object using the Euclidean distance. The end of a bout was defined as the time at which neither nose nor tail were within the interacting radius of the object (end of retreat). The start of retreat was set to be the timepoint when the nose was closest to the object during an interacting bout. The above-mentioned metrics of interacting radius as well as timepoints for exploration were based on prior studies ^13,18^.

#### Threat-reward conflict assay

The threat-reward conflict assay (“monster” assay) was performed similar to what is described previously ^20^. All animals were food restricted and handled a day before the behavioral assay started. On the first day, animals were allowed to habituate to an initial “shelter” with the doors closed for ~30 minutes. Animals were then trained for 2–4 days to collect rewards without the monster present. The mouse was first introduced into the shelter with the door closed. Doors to the shelter would then open and the animal was allowed to explore the open arena. If the mouse licked the spout, a single drop (10 μL) of Ensure was delivered. After the drop was delivered, further licking would not elicit more reward. The trial would then end once the animal returned into the shelter zone and the doors closed. The trial would also end if the animal did not leave the shelter for three minutes after the doors opened. After a 20 second inter-trial interval, the doors opened once again and the next trial began. Animals were allowed to perform 20 trials in a given day or until two hours passed. In between animals, the floor of the arena and the dispensing spout were thoroughly cleaned with 70% ethanol. Only animals which achieved at least 12 rewards across the 20 trials by the end of training progressed to the next stage.

After training all animals to collect rewards in the absence of the threat, half of the animals were refed overnight. Then, the following day, all animals were again allowed to explore the arena (as above) to collect rewards (day C1). After day C1, a large threatening object (“monster”; Jurassic World Velociraptor Blue 1/2 Mask, Rubies) was introduced at the far end of the arena on the following day (M1). As conducted in prior studies, for day M1, animals were allowed to leave the shelter and forage for caloric reward. However, once the animal moved past a pre-defined line from the shelter (30 cm, as defined by an IR beam break), the monster would lunge at the animal and emit a loud noise (120 dB, Godzilla Sounds, SoundBible.com, https://soundbible.com/tags-godzilla.htm) and repeatedly move back and forth. Importantly, the lunging of the monster would not overlap with the reward spout, but rather stopped just short of it, such that the animal would be able to collect caloric reward without being harmed.

#### Chocolate Pellet Exploration Assay

Experimentation was performed as described in Lodder et al., 2025 ^32^. Briefly, animals were food restricted at least one day prior to the beginning of behavioral experimentation. Animals were then placed into a small arena (8 in. × 8 in.) and allowed to habituate to the arena for 10 minutes. Animals were then delivered 10 pellets (F05301, Bio-Serv), one pellet a minute for 10 minutes, randomly in the arena. Animal movements were captured with the same overhead camera and recorded using Bonsai-rx. Manual video scoring was done to define when the animal retrieved the individual pellets. Data analyzed here is a subset of dataset found in Lodder et al., 2025 ^32^.

#### Stereotaxic surgical procedures

For all procedures, mice were anesthetized with inhaled isoflurane (1–3%) throughout the procedure and given *ad libitum* access to oral carprofen for a day before the operation. Under the stereotaxic frame (David Kopf Instruments), the skull was exposed and leveled and a small burr hole was drilled. AAVs that encoded DREADDs that required Cre for expression were injected initially in animals that did not express Cre recombinase to determine a concentration that showed minimal Cre-independent recombination and subsequent expression at the injection site. After burr hole creation, virus was injected at a rate of either 50 nL/min (TS) or 70 nL/min (SNc/SNpl, ARC) with a syringe pump (Harvard Apparatus, #883015). Pipettes were then allowed to rest at the injection site and above the injection site for at least 5 minutes each before they were slowly withdrawn (< 10 μm/sec) from the injection site. The following volumes (in nL) and coordinates were used for each site (represented as mm from bregma in the order AP, ML, DV):

TOS: 300 nL at −1.4, 3.28, −2.45

VLS: 100 nL at 0.5, 3.4, −3.25 with a 15° angle

ARC: 300 nL at −1.6, ±0.26, −5.85

SNc: 250 nL at −3.0, ±1.4, −4.0

SNpl: 250 nL at −3.1, ±2.1, −3.6 (for CRACM experiments, 150 nL was used)

For AAV injections alone, the skin was then sutured shut. For photometry recordings and optogenetic manipulations, the skull was then lightly scored with a razor blade to ensure adequate adhesion of implants. Animals were implanted with a fiber optic (MFC_200/230–0.37_3mm_MF1.25_FLT; Doric Lenses). For photometry recordings in particular, the implant was lowered to a depth of 150 μm above the virus injection site. The fiber was held in place with superglue (Loctite gel #454) and Metabond dental cement. For photometry experiments, recordings began at least three weeks post operation. For DREADD and optogenetic manipulation experiments, manipulations and recordings began at least four weeks post operation. For mapping of collaterals of TOS-projecting DANs, animals were perfused four weeks post operation.

AAVs and the concentrations (in gc/mL) used in this study were as follows:

AAV9-CAG-dLight3.8: 1.0 × 10^13^ (UNC Neurotools)

AAV9-hSyn-dLight3.8: 2.1 × 10^12^ (UNC Neurotools)

AAV9-hSyn-dLight3.8mut: 2.31 × 10^13^ (Plasmid design/modification made in-house, mutation and cloning done by Azenta Genewiz, packaged at UNC Neurotools. Plasmid available on Addgene)

AAV9-hSyn-mCherry: 1.1 × 10^13^ (Addgene #114472)

AAV9-hSyn-DIO-hM3D(G)q-mCherry: 1.1 × 10^13^ (Addgene #44361)

AAV8-hSyn-DIO-hM4D(G)i-mCherry: 2.1 × 10^13^ (Addgene #44362)

AAV8-hSyn-DIO-mCherry: 2.1 × 10^12^ (Addgene #50459)

AAV8-hSyn-FLEX-TC66T-P2A-EGFP-P2A-N2CG: 1.2 × 10^12^ (Plasmid designed in-house, cloned by Epoch Life Science and Azenta Genewiz, packaged at UNC Neurotools. Plasmid available on Addgene)

AAV8-Ef1α-FLEX-TVA-EGFP: 1.0 × 10^13^ (Salk Institute)

AAVDJ/9-nEF-Con/Foff 2.0-ChRmine-oScarlet: 8.7 × 10^12^ (Plasmid from Addgene #137161 and packaged at Janelia Viral Core)

AAVDJ/9-hSyn-FLEX-10xmyc: 5.0 × 10^12^ (Plasmid produced by Twist BioScience as in ^69^ and packaged at Janelia Viral Core)

AAVDJ/9-hSyn-FLEX-H2B-GFP: 1 × 10^13^ (Plasmid produced by Twist BioScience as in ^70^ and packaged at Janelia Viral Core)

AAVDJ/9-hSyn-DIO-mCherry: 5.6 × 10^12^ (Plasmid from Addgene #50459 and packaged at Janelia Viral Core)

AAVDJ/9-CAG-Cre (8 × 10^12^) (Plasmid produced by Twist Bioscience as in Yang et al., 2023^71^ and packaged at Janelia Viral Core)

AAV9-EF1a-DIO-hChR2(H134R)-eYFP-WPRE_HGHpA: 2.7 × 10^13^ (Addgene #20297)

#### Fiber photometry (intensity) recording

For intensity fiber photometry experiments, a fiber optic implant on each animal was connected with a 0.37 NA, 3 meters long low autofluorescence patchcord (MFP_200/220/900–0.37_3m_FCM-MF1.25, Doric Lenses) to a Doric minicube with integrated photodetector (iFMC5-G2_E1(460–490)_F1(500–540)_E2(555–570)_F2(580–680)_S, Doric Lenses). Excitation light was generated from blue (470-nm excitation light, M470F3, Thorlabs; LED driver LEDD1B, Thorlabs) and green (470-nm excitation light, M565F3, Thorlabs; LED driver LEDD1B, Thorlabs) LEDs modulated at 200 and 250 Hz, respectively. Excitation light was maintained at an average of 15–30 μW for blue and green light. Signals were amplified in DC mode in the integrated Doric minicube and collected by a data acquisition board (PCI-6115, National Instruments) at 4 kHz, controlled by ScanImage in MATLAB 2012a. The acquisition board also received a synchronizing pulse generated by the behavior system for alignment of behavioral and neural time series data.

#### Fiber photometry (intensity) signal analysis

Intensity fiber photometry signals were analyzed as described previously ^76^. Briefly, raw signals were first detrended using a rolling Z-score with a time window of 30 seconds (12000 samples). This initial detrending reduced artifacts of photobleaching of the fluorophore over the entire session. Detrended, frequency-modulated signal was then demodulated using the Python spectrogram function implemented from the SciPy package, with a window of 216 samples with an overlap of 108 samples, corresponding to a final sampling period of 27 ms. The power of the signal at the frequency band closest to the carrier frequency was taken as the demodulated signal. Finally, to quantify fluorescent transients as Z-scores, the signal was passed through an additional 30 second Z-score window. To synchronize this neural time series data with the behavioral data, a synchronizing pulse was sent from the Bonsai system recording the video to the data acquisition board logging neural time series data at the beginning and end of the session. Timepoints for each frame were interpolated within these synchronizing pulses and the closest timepoint in the demodulated (downsampled) photometry data was taken for each behavioral timepoint used for aligning and averaging photometry signals (approach start, retreat start, and retreat end). For comparisons of photometry signal between groups of animals, signals were averaged within each animal across interaction bouts, and then across animals within a group. To compare the retreat-associated TOS DA signal between groups, we analyzed the magnitude of the Z-scored response from 1 second before to 1 second after retreat start. We used a broad window to capture the entire magnitude of the TOS DA transient when animals retreat from the novel object.

#### Fluorescence lifetime photometry at high temporal resolution (FLiP-R) recordings

Fluorescence lifetime photometry was performed using a custom-built high-speed system ^32^. dLight3.8 was found to be a useful sensor for lifetime imaging as it displays fluorescence lifetime responses which are highly correlated to the known DA-dependent intensity responses. To further validate the specificity of the lifetime sensor, we generated a ligand-binding mutant version of the sensor (with a single point mutation in the binding pocket) which displays minimal intensity or lifetime fluctuations ^32,77^. We blocked the receptor using a DA type-1 receptor (D1R) antagonist, SCH23390, and showed a decrease in the lifetime fluctuations reported. As positive controls, we optogenetically bidirectionally manipulated DANs and recorded resulting lifetime changes in the according downstream striatal subregion and showed that the lifetime signal is modulated by putative changes in the ligand of interest (DA) concentration ^32^.

For recording from animals in this specific experiment, animals were connected with a patch cord (MFP_200/220/900–0.37_2m_FCM-MF1.25, Doric Lenses) to the above-described system and allowed to habituate to the patch cord, with the laser on, in their home cage, for two to three minutes. The light intensity was matched across animals at 12 μW. Synchronization of fluorescence lifetime recordings to behavioral systems was performed as above. The fluorescence lifetime signal was downsampled by a moving average to a 10 Hz signal except for the chocolate pellet exploration assay for which the signal was downsampled to 20 Hz.

#### FLiP-R signal analysis

For comparisons of photometry signal between groups of animals, signals were averaged within each animal across interaction bouts, and then across animals within a group. To compare the retreat-associated TOS DA signal between, we took the average lifetime signal from 1 second before to 1 second after the start of retreat (the same window used as in intensity fiber photometry signal analysis).

#### Optogenetic calibration of dopamine release in the tail of striatum

To calibrate the optogenetic stimulation of TOS-projecting DANs, animals were first exposed to ten 0.5 mA, 500 ms long, foot shocks in a white plexiglass box with a grid floor through which shocks were delivered (Med Associates #ENV-005A). Shocks were delivered through the grid floor spaced apart by 20–30 seconds (with the inter-shock interval randomly chosen from a uniform distribution.) We then, for each site, tested a number of optogenetic stimulation parameters by varying the frequency, pulse width, and power of optogenetic stimulation train, though each stimulation train was for a duration of 500 ms (to best match the release evoked by a 500 ms foot shock). A 500 ms pulse train with 5 ms pulse width of 5 mW delivered at 15 Hz was chosen as best matching the DA release profile in the TOS caused by a foot shock. Animals that were used for calibration of the optogenetic stimulation were not used in the novel object exploration behavioral task since repeatedly exposing animals to the foot shock prior to the spontaneous open-field behavioral study would alter their exploration behavior.

#### Optogenetic manipulations of dopamine release during novel object exploration

For cohorts in which we employed optogenetic manipulations of DANs, animals were handled and habituated to the arena as in photometry recordings. On the novel object exposure day, Bonsai-rx was used for closed loop optogenetic stimulation. In Bonsai, a roughly circular ROI was drawn around the object ~7 cm in radius (the same interacting radius as used for defining interacting time in DeepLabCut). The image was then thresholded such that the dark animal appeared white and the background (floor, walls, object etc.) all appeared black. To prevent the patch cord from being detected as the animal in the ROI, white tape was wrapped around the patchcord. The ROI was then calibrated such that if any part of the animal’s body fell within the defined ROI for greater than 100 ms (to prevent jitter in real-time tracking from spuriously causing laser pulses), a 1 Hz pulse was generated from an Arduino Uno. This 1 Hz pulse was sent to a Master 8 pulse generator which then generated a pulse pattern for optogenetic stimulation (500 ms pulse train consisting of a 15 Hz pattern of 5 ms pulses at 5 mW per pulse). This resulted in a 50% duty cycle of stimulation, to evoke individual DA pulses rather than a sustained increased baseline DA with continuous pulsed stimulation. This optogenetic stimulation parameter was fed into an acousto-optic modulator (AOM) placed after a 635 nm laser (OptoEngine). An AOM was used instead of directly modulating the laser using a BNC port since the AOM allows for noiseless sub-millisecond modulation of laser power with minimal ramping. A shutter was not preferred since loud noises are known to induce DA release in the TOS ^13,20^. After the novel object assay, animals were transferred to a feeding cage to assess feeding behavior as described above. After assessment of feeding behavior, animals were transferred to the FLiP-R system and the evoked change in lifetime in the TOS due to optogenetic stimulation was measured for each side separately. The magnitude of the fluorescence lifetime evoked by stimulation was calculated for both sides of the brain for an animal and averaged.

#### DREADD manipulations of AgRP neurons during novel object exploration

For cohorts in which we employed DREADD manipulations of AgRP neurons, the novel object behavioral assay differed from the above section. For these animals, alongside the normal handling and habituation, on all days leading up to the novelty session day, they were given an intraperitoneal (IP) injection of 0.9% sterile saline to habituate the animals to the injection itself. After injection, on arena habituation days, the animals were transferred back to their home cage for 30 minutes and then placed into the arena. On the novelty session day, all animals (control and DREADD animals) were injected IP with 0.2 mg/kg of deschloroclozapine (DCZ) and then transferred to an empty (recovery) cage without food present. After 30 minutes, animals were transferred to the arena with the novel object present. After the novel object assay, animals were transferred to a feeding cage as described above, to measure feeding changes induced by AgRP manipulation.

#### Rabies tracing and CRACM surgical procedures

For rabies tracing without associated electrophysiology recordings, animals were first injected with a Cre-dependent AAV either with a bicistronic construct encoding both a weakened TVA (TC66T) and G protein (N2C(G)) or a Cre-dependent AAV with TVA-GFP alone in the SNc and SNpl. A burr hole was made in the same surgery over the TOS, and the surgical site was closed with sutures. After waiting 4 weeks to allow for sufficient expression of the helper proteins, the surgical site was reopened and EnvA-pseudotyped CVS-N2C-RbV-ΔG-H2b-tdTomato (4E9 pg/mL) was injected in the TOS. After waiting six days to allow for the rabies to infect axons and move retrograde transsynaptically, animals were perfused and histological sections were taken, as described below.

For CRACM experiments, the above rabies tracing surgical protocol was modified slightly. Animals were injected with three viruses at the first surgical timepoint: (1) a Cre-dependent AAV encoding a blue-light activated channelrhodopsin with a YFP tag at the ARC, (2) a Cre-dependent AAV with a bicistronic construct encoding TC66T and N2C(G) at the SNpl, and (3) an AAVDJ/9 encoding Cre recombinase. The surgical site was then closed with sutures. After waiting 4.5 weeks to allow for expression of the Cre recombinase in all TOS-projecting neurons and Cre-mediated recombination and subsequent expression of helper proteins in TOS-projecting SNpl neurons and Cre-mediated recombination and expression of the channelrhodopsin construct at the ARC in AgRP neurons, the surgical site was reopened. At this second timepoint, animals were injected with CVS-N2C-RbV-ΔG-H2b-tdTomato (4E9 pg/mL) in the SNpl and the surgical site was once again closed with sutures. After waiting five days to allow for the rabies virus to infect the TC66T+ neurons and move retrograde transsynaptically, animals were sacrificed and slices were collected for electrophysiology recordings outlined below.

#### Electrophysiology

Brain slices were obtained from 4-month-old mice roughly 5 weeks after the first injection. Mice were anaesthetized by isoflurane inhalation and perfused trans-cardially with ice-cold ACSF containing (in mM) 125 NaCl, 2.5 KCl, 25 NaHCO3, 2 CaCl2, 1 MgCl2, 1.25 NaH2PO4, and 25 glucose (295 mOsm/kg). Coronal slices of 250 μm thickness were obtained using a Leica VT1000 S vibratome in ice-cold ACSF beginning roughly 1 mm caudal to bregma to the most posterior section obtainable from the brain. The slices were then transferred to a holding chamber containing choline-based solution (consisting of (in mM): 110 choline chloride, 25 NaHCO3, 2.5 KCl, 7 MgCl2, 0.5 CaCl2, 1.25 NaH2PO4, 25 glucose, 11.6 ascorbic acid, and 3.1 pyruvic acid) at 34°C for 20–23 min then transferred to a secondary holding chamber containing ACSF and maintained at room temperature (20–22°C) until use. Slices were covered with aluminum foil while in the secondary chamber until transferred to electrophysiology rig. All recordings were obtained within 6 hours of slicing. Both choline solution and ACSF were bubbled with 95% O2/5% CO2.

During recordings, coronal slices were maintained in ACSF at 34 °C. Slices were transferred to a recording chamber and imaged with an upright microscope (Olympus BX51WI) through a 60x water-immersion objective. For whole-cell recordings, patch pipettes were pulled from borosilicate glass (G150F-3, Warner Instruments) to a resistance of 2–4 MΩ and filled with low Cl^−^ internal solution containing (in mM), 135 CsMeSO3, 10 HEPES, 1 EGTA, 3.3 QX314-Cl, 4 Mg-ATP, 0.3 Na-GTP, 8 Na2-phosphocreatine (pH 7.3 adjusted with CsOH; 295 mOsm/kg). The dimmest RbV+ were selected since these cells seemingly showed the best cell health as assessed by morphology in the densest parts of the axonal projection field. Whole-cell recordings were conducted in voltage-clamp mode, first at 0 mV to record evoked IPSCs, and then for a subset of cells we recorded at −70 mV to record potentially evoked EPSCs. For each cell, at least 10 sweeps/recordings were performed, with each recording being 10 seconds long with a 10 second inter-sweep interval. One second into the sweep, a short (3 ms) pulse of full field illumination (6 mW) was delivered by a blue laser (OptoEngine; 473 nm) controlled through an AOM. Thus, roughly there was a wait of ~20 seconds between each stimulation to allow for recovery.

#### Histology and Immunohistochemistry

Mice were anesthetized with high concentration isoflurane inhalation and trans-cardially perfused with phosphate-buffered saline (PBS) and subsequently 4% paraformaldehyde (PFA) in PBS. Brains were then extracted and stored in 4% PFA/PBS overnight and then transferred to PBS for long-term storage. Brains were sliced using a Leica VT1000s vibratome into 70 μm thick free-floating sections. Slices were transferred to a six well plate and washed with PBS. Slices were then blocked at room temperature for two hours in 5% normal goat serum (Abcam), 0.1% TritonX-100 PBS. Slices were then placed into a solution with primary antibody (all at 1:1000) and allowed to incubate overnight at 4 ˚C. The following day, slices were washed with PBS with 0.1% TritonX-100 (PBST). Slices were then transferred to a solution with secondary antibody at 1:500 in blocking buffer. Slices were washed finally with PBST and placed in PBS before being mounted on a slide for imaging with ProLong Diamond Antifade Mountant with DAPI (ThermoFisher Scientific). Slices were imaged with an Olympus VS200 slide scanning microscope or for high-resolution images, a Yokagawa CSU-X1 spinning disk confocal microscope.

### QUANTIFICATION AND STATISTICAL ANALYSIS

All data was analyzed using Python v3.8 and statistical tests performed using the pingouin package (a wrapper around many SciPy statistical functions). No statistical method was used to pre-determine sample size, though we attempted to match sample size across experiments. For DREADD and optogenetic manipulation experiments, experimenters were blinded to the animal condition. Experimenters were not blinded to the animal’s food restriction status given the need for placing special cage cards designating food restriction status. Non-parametric, two-sided statistical tests were used for all analysis and statistical significance was taken as p < 0.05, save for comparison of correlation coefficients to an assumed population mean of zero. When necessary (and as indicated), Bonferroni correction for multiple comparisons was employed. In figures, n/s:p>.05, *:p<.05, **:p<.01, ***:p<.001, ****:p<.0001. Data was not systematically tested for normalcy, hence the use of non-parametric statistics. All bar and point graphs are shown as the mean with error bars as the standard error about the mean (SEM). In boxplots, edges of the box are the 25^th^ and 75^th^ percentile, with whiskers extending to the most extreme data points not considered an outlier. For regression analysis, Pearson’s correlation or Spearman’s rank correlation were used. For all experiments, if an animal displaced the block from the floor of the arena, the data was not used (n=1). For all experiments, if an animal displayed significant tangling in the patch cord around the novel object block, the behavioral and photometry data was not used (n=1). For refeeding experiments, animals that removed the food out of the food bowl (making it difficult to measure amount eaten), were not included in feeding analysis but were included in the rest of the behavioral analysis (n=1).

## Supplementary Material

1

## Figures and Tables

**Table:** KEY RESOURCES TABLE

REAGENT or RESOURCE	SOURCE	IDENTIFIER
Antibodies		
Rabbit polyclonal anti-dsRed	Takara	RRID:AB_10013483;Cat. #632496
Chicken polyclonal anti-GFP	Abcam	RRID:AB_300798;Cat. #13970
Goat polyclonal anti-AgRP	Thermofisher Scientific	RRID:AB_2608453; Cat. #PA5–47831
Mouse anti-tyrosine hydroxylase	Immunostar	RRID:AB_572268; Cat. #22941
		
Bacterial and virus strains		
AAV9-CAG-dLight3.8	UNC Neurotools	AAV in stock
AAV9-hSyn-dLight3.8	UNC Neurotools	AAV in stock
AAV9-hSyn-dLight3.8mut	This paper	To be deposited to Addgene
AAV8-hSyn-mCherry	Deisseroth Lab	RRID:Addgene_114 472; #114472-AAV8
AAV9-hSyn-DIO-hM3D(G)q-mCherry	Addgene	RRID: Addgene_44361; #44361-AAV9
AAV8-hSyn-DIO-hM4D(G)i-mCherry	Addgene	RRID: Addgene_44362; #44362-AAV8
AAV8-hSyn-DIO-mCherry	Addgene	RRID: Addgene_50459; #50459-AAV8
AAV8-hSyn-FLEX-TC66T-P2A-EGFP-P2A-N2CG	This paper	To be deposited to Addgene
AAV8-Ef1a-FLEX-TVA-EGFP	Salk Institute	26198
pAAV-nEF-Con/Foff 2.0-ChRmine-oScarlet	Addgene	RRID: Addgene_137161; #137161
pAAV-hSyn-FLEX-10xmyc	Capelli et al., 2017^69^	
pAAV-hSyn-FLEX-H2B-GFP	Ruder et al., 2021^70^	
pAAV-CAG-Cre	Yang et al., 2023^71^	
EnvA-pseudotyped CVS-N2C-RbV-ΔG-H2b-tdTomato	Janelia Viral Core	
		
Chemicals, peptides, and recombinant proteins		
Deschloroclozapine	MedChem Express	Cat. #HY-42110
		
Deposited data		
Python analysis code	This paper	To be deposited to GitHub
Video tracking and dopamine photometry data	This paper	To be deposited to Zenodo
		
Experimental models: Organisms/strains		
Mouse: C57BL/6J	Jackson Laboratory	RRID: IMSR_JAX:000664
Mouse: AgRP-ires-Cre	Jackson Laboratory	RRID: IMSR_JAX:012899
Mouse: DAT-ires-Cre	Jackson Laboratory	RRID: IMSR_JAX:006660
		
Software and algorithms		
Motion Sequencing	Wiltschko et al., 2015^22^	https://dattalab.github.io/moseq2-website/index.html
DeepLabCut	Mathis et al., 2018^25^	https://github.com/DeepLabCut/DeepLabCut
MATLAB	Mathworks	https://www.mathworks.com/; RRID:SCR_001622
Python		https://www.python.org/downloads/;
ScanImage (2012a version)	Bernardo Sabatini	https://github.com/bernardosabatini/SabalabAcq
ScanImage (2021a version)	Bernardo Sabatini	https://github.com/bernardosabatini/SabalabAcq
Custom MATLAB Scripts (fluorescence lifetime data acquisition)	Lodder et al., 2025^32^	
Python	https://www.python.org/	https://www.python.org/
Bonsai-rx	Lopes et al., 2015^38^	https://bonsai-rx.org/ ; RRID:SCR_021512
Thorlabs PMT Controller	Thorlabs	https://www.thorlabs.com/software_pages/ViewSoftwarePage.cfm?Code=PMT
Pulsed laser controller	Becker & Hickl	https://www.becker-hickl.com/products/dcc-100-dcc-100pcie/
		
Other		
Novelty object: Mega Blok	Mega Blok	Cat.# DCH63
Lighting and MoSeq Novelty Arena + Camera Acquisition setup	Akiti et al., 2022^18^	
White novelty arena flooring/walls	Canal Plastics Center	Cat.#7508
White novelty arena camera	Teledyne Vision Solutions	Cat.# FL3-U3–13E4M
Nature’s Miracle Enzymatic Cleaner	Nature’s Miracle	https://www.naturesmiracle.com/
Chocolate Pellets	BioServ	Cat.#: F05301
Pellet Dispenser	MedAssociates	Cat.#: ENV-203–20
Ensure (Vanilla flavor)	Ensure	https://www.ensure.com/nutrition-products/ensure-original/vanilla-shake
Monster Task Arena + Setup	Tsutsui-Kimura et al., 2025^20^	
Blue pulsed laser	Becker & Hickl	Cat.# BDS-SM-473-FBC
Custom analog processing unit	Lodder et al., 2025^32^	
Optical dichroic filter	Semrock	Cat.# Di02-R48825x36
Interference filter	Idex HS	Cat.# FF01–525/35–25
GaAsp Photomultiplier Tube	Thorlabs	Cat.#: PMT2101
Fiber implants	Doric Lenses	Cat.# MFC_200/230–0.37_3mm_MF1.25_FLT
Blue fiber-coupled LED	Thorlabs	Cat.#M470F4
Green fiber-coupled LED	Thorlabs	Cat.#M565F3
Fiber-coupled LED driver	Thorlabs	Cat.#LEDD1B
Fiber optic patch cord	Doric Lenses	MFP_200/220/900–0.37_3m_FCM-MF1.25
Minicube with integrated photodetector	Doric Lenses	iFMC5-G2_E1(460–490)_F1(500–540)_E2(555–570)_F2(580–680)_S
Data acquisition board	National Instruments	PCI-6115
Footshock apparatus	MedAssociates	Cat. #ENV-005A
Master 8 Pulse Generator	MicroProbes for Life Sciences	RRID#: SCR_018889
635 nm laser	OptoEngine	Cat.#MRL-III-635L
Acousto-optic modulator	Brimrose	Model No.: TEM-80–2-473 / 532
AOM Radio Frequency Driver	Brimrose	Model No.: FF-80-B1-B2-V2

## Highlights

- Hunger restructures behavior and increases directed exploration of a novel object
- Hunger suppresses dopamine in the tail of striatum (TOS) to increase exploration
- Activity of AgRP neurons encodes the hunger signal which suppresses TOS dopamine
- AgRP neurons are connected to the TOS through the lateral hypothalamus
