# Supplementary material for: Hunger modulates exploration through suppression of dopamine signaling in the tail of striatum
Source: Neuron. Author manuscript; Available in PMC 2025 Dec 18. (PMC12712896; doi:10.1016/j.neuron.2025.09.009)
Supplement: 1 [file NIHMS2111955-supplement-1.pdf]

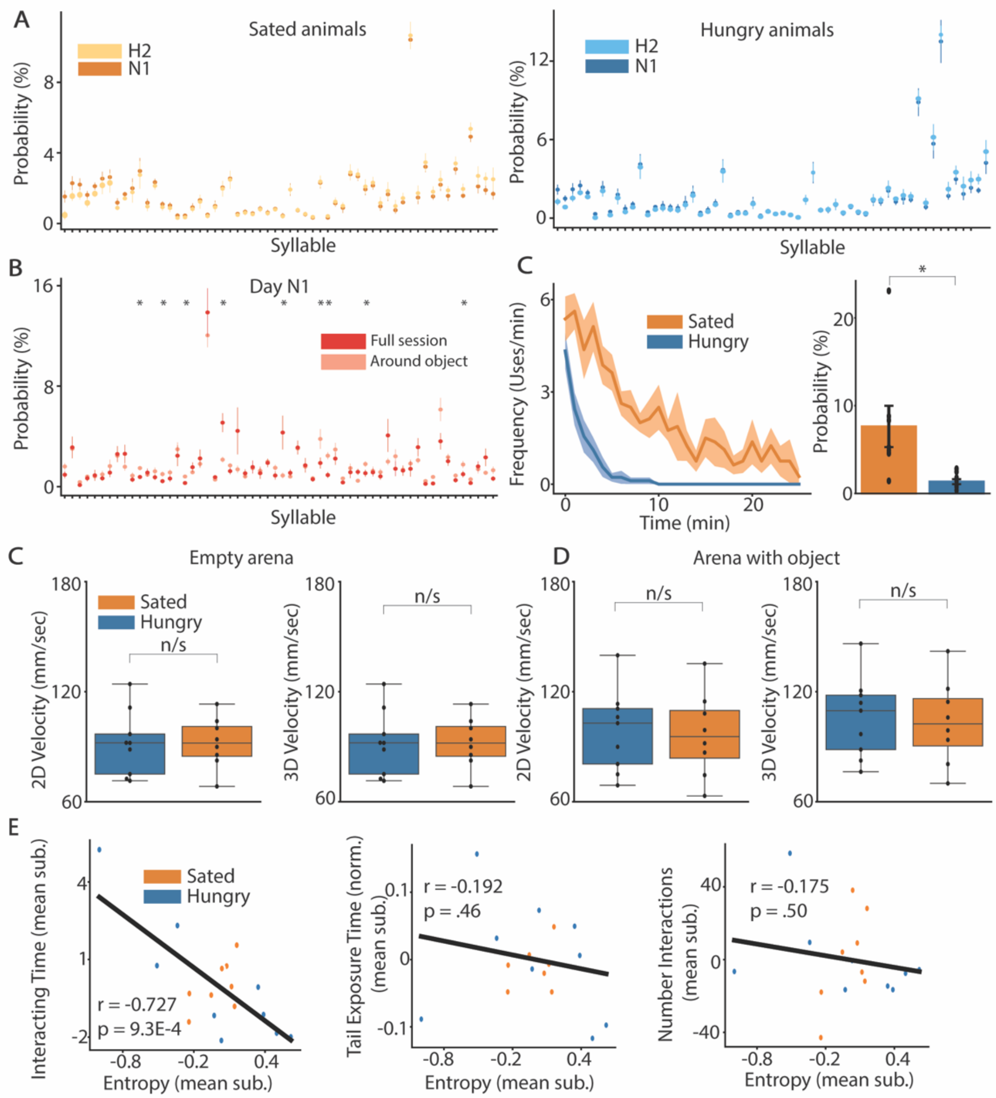

Figure S1: Further analysis of behavioral changes in hungry and sated mice in novel object exploration assay related to Figures 1 and 2

- **A)** Comparison of syllable usage in sated (left) and hungry (right) mice between the second day of habituation to the open arena (H2) and the first day of novel object exposure (N1). Wilcoxon signed-rank test used with Bonferroni correction.
- **B)** Comparison of syllable usage when animals are near the object versus syllable usage during the entire session (N1). Wilcoxon signed-rank test used with Bonferroni correction.
- **C)** Comparison of dynamic usage of syllable 50 over the entire session in hungry versus sated animals (left) as well as comparison of the overall proportion of usage of syllable 50 relative to other syllables when hungry and sated animals are near the novel object on day N1.
- **D)** Comparison of two-dimensional (left; U=34, p=0.888) and three-dimensional (right; U=34, p=0.888) velocity of hungry and sated mice, averaged within mouse across only habituation sessions.

E) As S1A-B, except for days with the novel object present (two-dimensional: U=38, p=0. 888; three-dimensional: U=38, p=0. 888)

F) Regression (Pearson's correlation coefficient) of the mean subtracted novel object exploration metric (left: Interacting time, middle: Tail exposure time normalized, right: Number of interactions) and mean subtracted syllable transition entropy across animals on day N1.

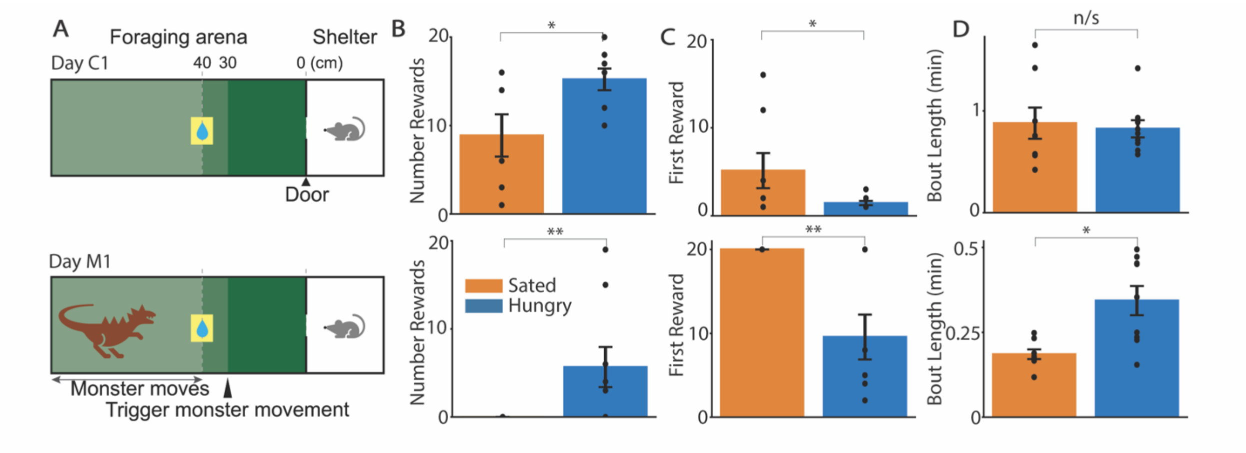

Figure S2: Hunger modulation of behavior in a threat-reward conflict assay related to Figure 2

- **A)** Schematic of the threat-reward conflict assay. Adapted from Tsutsui-Kimura et al., 2025[20].
- **B)** Number of rewards mice acquired on day without monster present (top) or with monster present (bottom) for hungry and sated animals.
- **C)** As in panel B, except comparing trial at which animal first collects reward.
- **D)** As in panel B, except comparing average amount of time animal spends in the foraging arena in a given bout.

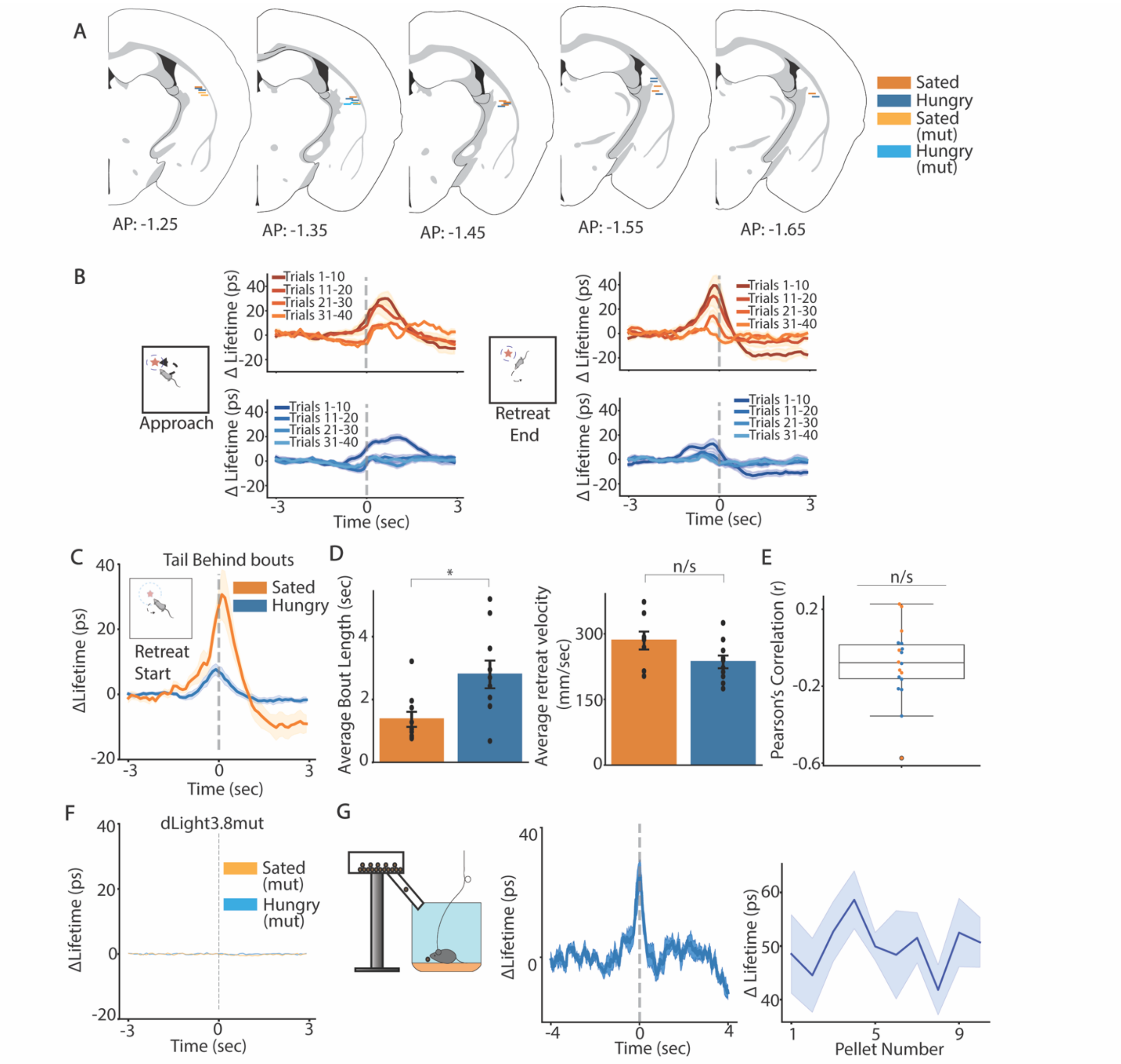

Figure S3: Schematic of fiber placements in TOS for Figure 3, and comparison of TOS DA changes due to ligand-independent effects/gross behavioral differences, and TOS DA modulation to an unexpected chocolate pellet related to Figure 3

- **A)** Schematic of fiber tip placements for dLight3.8 lifetime recordings in the TOS. Each bar represents a fiber optic tip.
- **B)** Difference in dLight3.8 fluorescence lifetime fluctuations as animals make repeated interactions with the object ("trials") aligned to approach towards the object (left) and aligned to end of interaction with object (right).
- **C)** Comparison of dLight3.8 lifetime signal on trials in which both hungry and sated animals retreated from the object with tail behind the entire time. Bold line represents mean and shaded area is SEM calculated across animals.
- **D)** Comparison of average bout length (left), and average retreat velocity (right), between hungry and sated animals.

E) Boxplot of regression coefficients of retreat velocity (bout-by-bout) with TOS DA response on a given bout across hungry and sated mice. One-sample t-test used to compare regression coefficients to 0.

F) Comparison of changes in dLight3.8mut lifetime signal in hungry and sated animals retreating form a novel object (U=6, p=0.7; n=3 sated, n=3 hungry animals.)

G) Chocolate pellet exploration assay schematic (left), TOS DA modulation when animals approach and retrieve the pellet (middle) and comparison of magnitude of TOS DA signal across multiple pellet retrievals (right). Data analyzed here is a subset of data shown in Lodder et al., 2025 [32]**.**

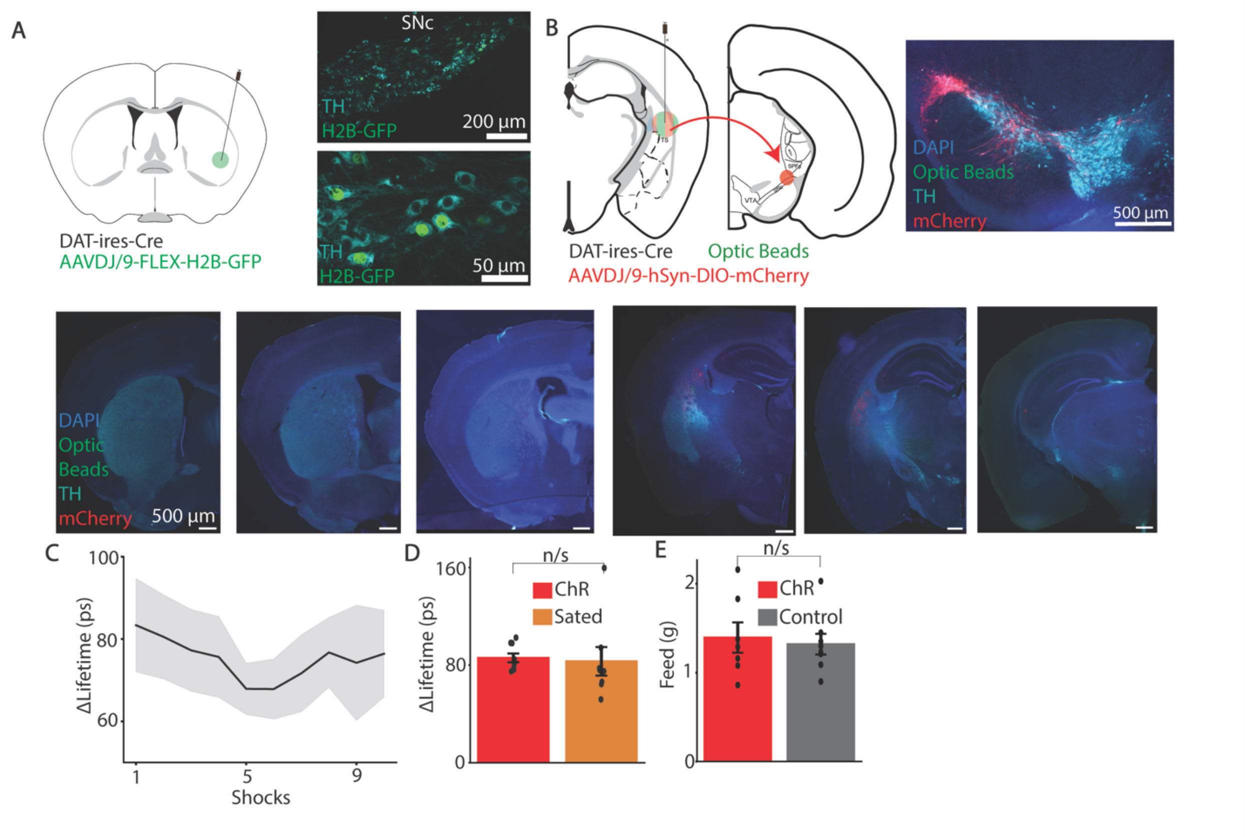

Figure S4: Validation of use of AAVDJ/9 for manipulating DA signaling in the TOS related to Figure 4

- **A)** Injection schematic (injection of AAVDJ/9 virus in the ventrolateral striatum, VLS) and histological confirmation of AAVDJ/9 mediated retrograde infection of TH+ neurons in the midbrain (right).
- **B)** Injection schematic (injection of AAVDJ/9 virus in the TOS) and histological confirmation of AAVDJ/9 mediated retrograde infection of TH+ neurons in the midbrain, primarily in the SNpl (top). Example histology of axonal arborization of these TOS-projecting DANs (bottom).
- **C)** Comparison of foot shock-evoked TOS DA response over repeated shocks within a given day.
- **D)** Comparison of optogenetically evoked DA release in the TOS with maximum TOS DA signal observed in sated animals during first ten interactions with the novel object.
- **E)** Comparison of feeding between animals transfected with opsin (ChRmine) and animals transfected with control non-opsin protein (10xmyc).

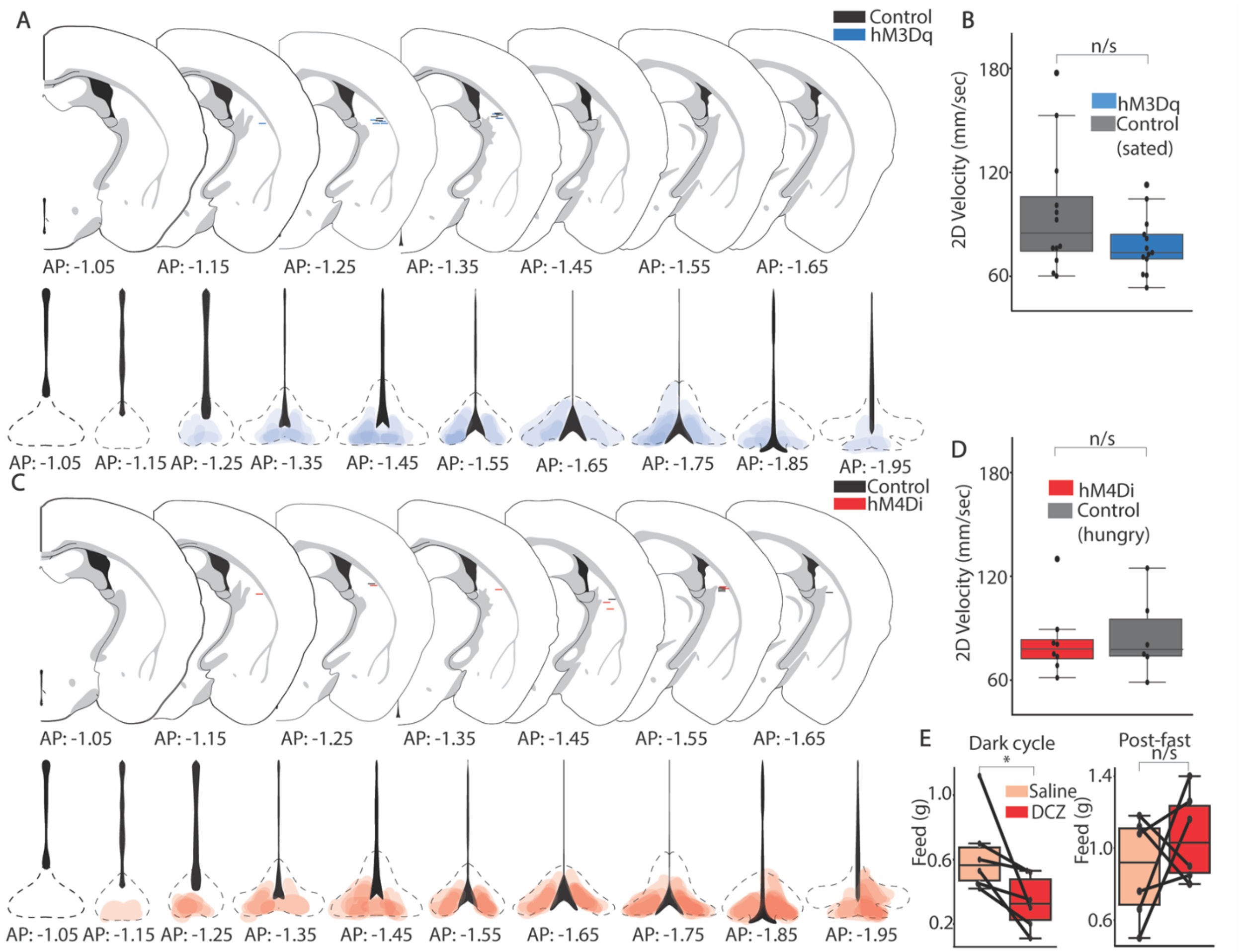

Figure S5: Schematic of DREADD AAV viral spread and photometry fiber tip locations in ARC and TOS, respectively and effect of DREADD mediated inhibition of AgRP neurons on dark-cycle feeding versus post-fast refeeding related to Figure 5

- **A)** TOS fiber placements and contour map of hM3Dq viral spread
- **B)** Comparison of two-dimensional velocity of hM3Dq versus control sated mice
- **C)** TOS fiber placements and contour map of hM4Di viral spread
- **D)** Comparison of two-dimensional velocity of hM4Di versus control hungry mice
- **E)** Comparison of feeding changes with DCZ versus saline injection in sated hM4Di mice tested at the beginning of the dark cycle (left) and food-restricted hM4Di mice tested during the middle of the dark cycle (right; Wilcoxon signed rank test, W=7, p=0.563).

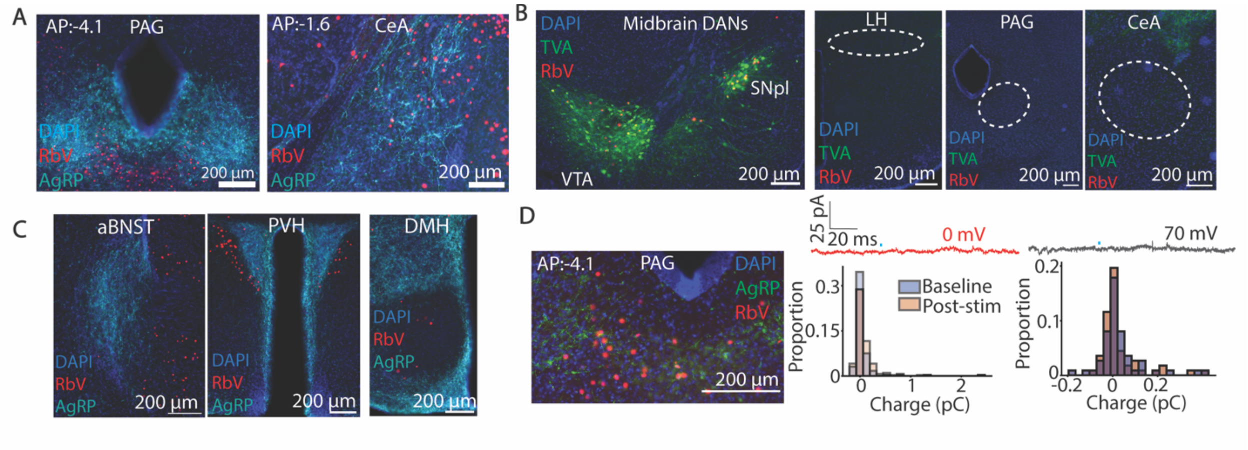

Figure S6: Extended rabies tracing results of the link between AgRP neurons and the TOS related to Figure 6

- **A)** Overlap between AgRP axons and RbV+ in other major AgRP projection fields – periaqueductal grey (left) and central amygdala (right).
- **B)** RbV+ neurons (red) at midbrain DANs of DAT-*ires*-Cre animals injected with cre-dependent AAV encoding TVA alone (green) (left) and lack of RbV+ neurons at LH, CeA, and PAG in same animal. Dashed white lines represent areas of AgRP+ axons and RbV+ neurons in animals that have both the TVA and G protein present.
- **C)** Minor overlap between AgRP axons and RbV+ neurons in several major AgRP projection fields (anterior bed nucleus stria terminalis, aBNST; paraventricular hypothalamus, PVH; dorsomedial hypothalamus, DMH)
- **D)** Whole-cell recordings from RbV+ neurons in the PAG (left, histological panel) held at 0 mV (middle) and -70 mV (right).

Table S1. Syllable labels for hungry and sated animals analyzed in Motion Sequencing, with p-values for statistical comparisons related to Figure 1.

Syllable ID	Associated Behavior	p-value for comparison of syllable usage between hungry and sated animals in empty arena	p-value for comparison of syllable usage between hungry and sated animals in object arena	p-value for comparison of syllable usage within hungry animals between H2 and N1	p-value for comparison of syllable usage within sated animals between H2 and N1	p-value for comparison of syllable usage around the object between sated and hungry animals on N1
2	Walking	1	1	1	1	0.458435
4	Rear	**0.004772**	**0.004772**	1	1	1
8	Pause	1	1	1	1	0.385864
9	Crouch	1	1	1	1	1
14	Rear	**0.004772**	**0.036577**	1	1	1
17	Walking	1	1	1	1	1
19	Rear	**0.009543**	**0.004772**	1	1	1
20	Pause	1	1	1	1	1
21	Walk	0.458083	0.143151	1	0.453125	1
22	Down from rear	1	1	1	1	1
23	Run	1	1	1	1	**0.048676**
24	Walk	1	1	1	1	1
25	Pause	0.143151	**0.033402**	1	1	1
26	Stop during walk	1	1	1	1	**0.044250**
27	Rear	**0.004772**	**0.036577**	1	1	1
28	Dart	**0.009543**	**0.004772**	1	1	1
30	Run	1	1	1	1	**0.048676**
31	Scrunch	1	1	1	1	1
32	Rear down	1	0.882764	1	1	1
33	Pause	0.090662	1	1	1	1
34	Walk	1	1	1	1	1
36	Crouch down	1	1	1	1	**0.038055**
38	Dart	**0.009543**	**0.033402**	1	1	1
40	Crouch down	0.05726	0.05726	1	0.906250	0.385864
43	Turn	0.319704	0.05726	1	1	1
45	Extend	1	1	1	1	1
46	Pause	0.05726	0.214726	1	1	0.323029
48	Run	1	1	1	1	1

49	Walk	1	1	1	1	0.385864
50	Approach/at tend	1	**0.009543**	0.226562	0.453125	**0.000885**
51	Slight turn	0.882764	0.214726	1	1	1
52	Slight rear	1	1	1	1	1
53	Scrunch	1	1	0.226562	1	1
59	Pause	0.214726	0.882764	1	1	1
60	Slight pause	0.319704	0.05726	1	1	**0.016815**
61	Crouch down	1	1	1	1	**0.001770**
68	Pause against wall	1	1	1	1	0.458435
69	Scrunch	1	1	1	1	1
70	Extend	**0.019087**	**0.004772**	1	1	1
71	Rear	1	1	1	1	0.458435
72	Pause	1	1	1	1	**0.016815**
73	Slight rear	0.143151	0.143151	0.67968	0.906250	1
74	Rear	**0.004772**	**0.004772**	1	1	1
77	Pause	1	1	1	1	1
78	Walk	0.143151	0.214726	1	1	0.183197
79	Wall rear	0.143151	0.090662	1	1	1
81	Attend with head up	0.458083	0.214726	1	1	1
82	Turn slightly	1	1	1	1	1
84	Pause mid-walk	1	1	1	1	1
85	Dart	1	1	1	1	0.223022
88	Turn slightly	**0.004772**	**0.004772**	1	1	0.121246
90	Walk	1	1	1	1	1
91	Retract head	0.458083	0.319704	1	1	0.869965
93	Slight rear	**0.004772**	0.644179	1	1	**0.016815**
96	Slight rear	**0.033402**	0.644179	1	1	1
97	Pause	1	1	1	1	1
98	Waddle	1	1	1	1	1
99	Extend	**0.004772**	**0.004772**	1	1	1
